# Deciphering the role of polyphenol in defence mechanism against tea mosquito bug (*Helopeltis theivora* Waterhouse.) in cocoa (*Theobroma cocoa* L.)

**DOI:** 10.1371/journal.pone.0271432

**Published:** 2022-10-14

**Authors:** Shilpa K. S., Minimol J. S., Gavas Rakesh, Suma B., Jiji Joseph, Maheswarappa H. P., Panchami P. S.

**Affiliations:** 1 Department of Plant Breeding and Genetics, College of Agriculture, Kerala Agricultural University, Thrissur, Kerala, India; 2 Plant Breeding and Genetics, Cocoa Research Centre, Kerala Agricultural University, Thrissur, Kerala, India; 3 Agricultural Entomology, Banana Research Station, Kerala Agricultural University, Thrissur, Kerala, India; 4 Cocoa Research Centre, Kerala Agricultural University, Thrissur, Kerala, India; 5 University of Horticultural Sciences, Bagalkot, Karnataka, India; Universita degli Studi della Basilicata, ITALY

## Abstract

Tea mosquito bug (TMB) is a serious pest of cocoa whose prevalence is high, mostly during summer and post monsoon season. Three species of tea mosquito bug have been reported on cocoa: *Helopeltis antonii* Signoret, *H*. *theivora* Waterhouse, and *H*. *bradyi* Waterhouse. *H*. *theivora* is the most prevalent one causing damage to young shoots, cherelles and pods. Rearing of tea mosquito bug on cocoa was found to be a failure in the present study hence *Helopeltis theivora* Waterhouse was maintained on the alternate host mile-a-minute (*Mikania micrantha Kunth*) under laboratory condition in insect rearing cages. Using freshly reared tea mosquito bugs twenty cocoa hybrids were screened for resistance and were ranked after 72 hours of screening. All the hybrids having less than three lesions per plant in seedlings and less than 33 lesions on pods were ranked as highly resistant. It was observed that hybrids classified as highly resistant had significantly higher phenol content than those classified as susceptible. The significantly low phenol content in the susceptible hybrids suggests that phenolics have a function in mediating resistance to tea mosquito bug in cocoa. From correlation and regression analysis it is confirmed that phenol content can be used as a potential marker indicating the level of resistance of cocoa hybrids against tea mosquito bug resistance.

## Introduction

Insects belonging to the family Miridae are serious pests of cocoa (*Theobroma cacao* L.) worldwide [1]. Tea mosquito bug (TMB), is a significant mirid pest of cocoa, whose prevalence has increased seriously during the most recent couple of years in summer and post monsoon season. Three species of tea mosquito have been reported on cocoa: *Helopeltis antonii* Signoret, *H*. *theivora* Waterhouse, and *H*. *bradyi* Waterhouse. *H*. *theivora* is the most prevalent one causing damage to young shoots, cherelles and pods [2]. Feeding lesions produced by tea mosquito bug can kill small cherelle, while older pods can continue to grow even if badly injured [3]. However, the yield is harmed as a result of malformed pods [4]. The cocoa pollinator (*Forcipomyia* spp.) and the pest both are the members of the Miridae family. As a result, chemical control becomes more complicated. Insecticides used to reduce tea mosquitos also limit the pollinator population, resulting in considerable crop losses. The development and deployment of tea mosquito bug resistant cocoa genotypes is the greatest alternative to chemical control [5]. Identification of a morphological marker is a crucial step in confirming resistance. Plant polyphenols are secondary metabolites, and they are one of the most abundant compounds in plants. Polyphenols are thought to have a key function in imparting resistance to many insect pests [6]. Hence this investigation was carried out with the goal of identifying tea mosquito-resistant cocoa genotypes and determining the effect of polyphenol in imparting resistance.

## Materials and methods

Twenty cocoa hybrids were evaluated for tea mosquito resistance in Cocoa Research Centre, Kerala Agricultural University. Details of hybrids are presented in Table 1.

**Table 1:** List of hybrids selected for tea mosquito bug screening.

Sl. no.	Hybrids	Parentage
1.	PIV 45.4	GI5.9 x GI10.2
2.	PIII 2.3	H7.1 x H5.3
3.	PIV 59.8	H10.1 x H6.8
4.	SIV 10.11	GI5.9 x GI10.2
5.	VSDI 10.13	GIV126 x GIV18.5
6.	SIV 1.10	GIV_68_ x GI_5.9_
7.	PIV 60.9	GII20.4 x GI5.9
8.	PII 12.11	GIV_24_ x GIV_51_
9.	SIV 5.15	GII20.4 x GI5.9
10.	VSDI 33.4	GIV148 x GIV18.5
11.	VSDI 23.21	GIV171 x GIV18.5
12.	PIV 58.6	GII20.4 x GI5.9
13.	PIV 56.9	GIV148 x GIV18.5
14.	VSDI 30.8	GIV18.8 x GIV18.5
15.	VSDI 11.11	GVI126 x GIV18.5
16.	SIV 1.6	GVI_51_ x GI_5.9_
17.	PIV 19.9	H5.3 x H6.1
18.	PIV 26.8	H7.10 x H3.5
19.	PIII 15.9	H10.1 x H6.8
20.	VSDI 29.9	GVI_188_ x GVI_55_

### Collection and rearing of TMB (*Helopeltis theivora* Waterhouse.)

The initial culture of tea mosquito bug was established with collections from the farm of Cocoa Research Centre. Adult male and females were collected from the field during morning hours. Attempt to rare it on cocoa seedlings was a failure due to lack of oviposition in cocoa. The alternate host mile-a-minute (*Mikania micrantha*) was utilised to combat the problem. A standard procedure was followed for rearing TMB on alternate host [7]. Newly reared insects collected from the raring cage were used for further screening procedure.

### Screening of tea mosquito bug on cocoa seedlings

**Fig 1:**
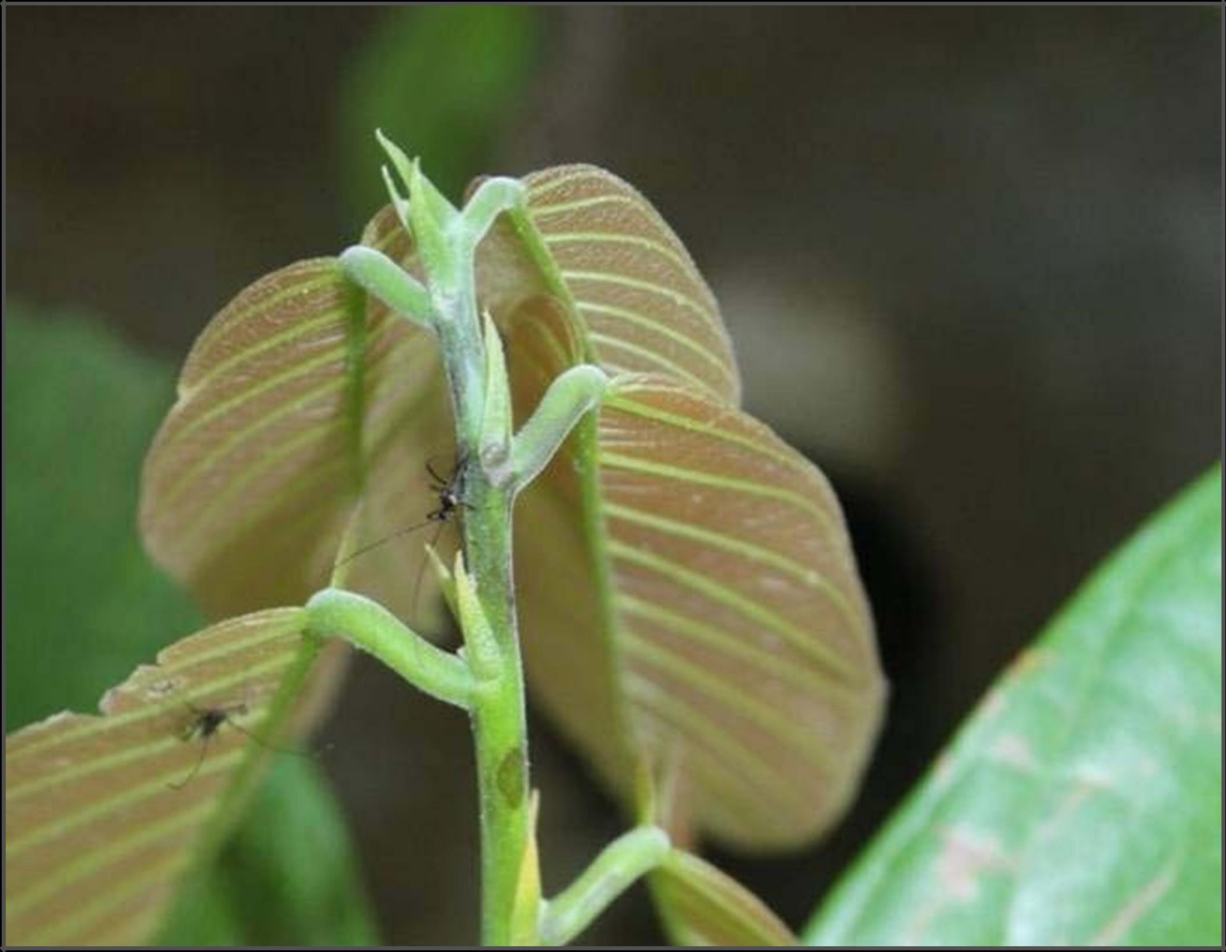
Screening on budded plants.

Patch budding was done on six-month-old root stock to raise the screening materials. Budded plants were brought up in the nursery. Screening was done on six-month-old budded plant. Three replications having five budded plants of each hybrid were screened inside the insect net house facility. Freshly reared *Helopeltis theivora* were introduced into the insect net house at a rate of 100 adults (50 male and 50 female) for every screening test. The severity of the infestation on the shoots/leaves was measured by counting the number of feeding lesions (Fig 1) for every 12 hours interval for 72 hours and then scoring it [8].

### Screening of tea mosquito bug on pods

**Fig 2:**
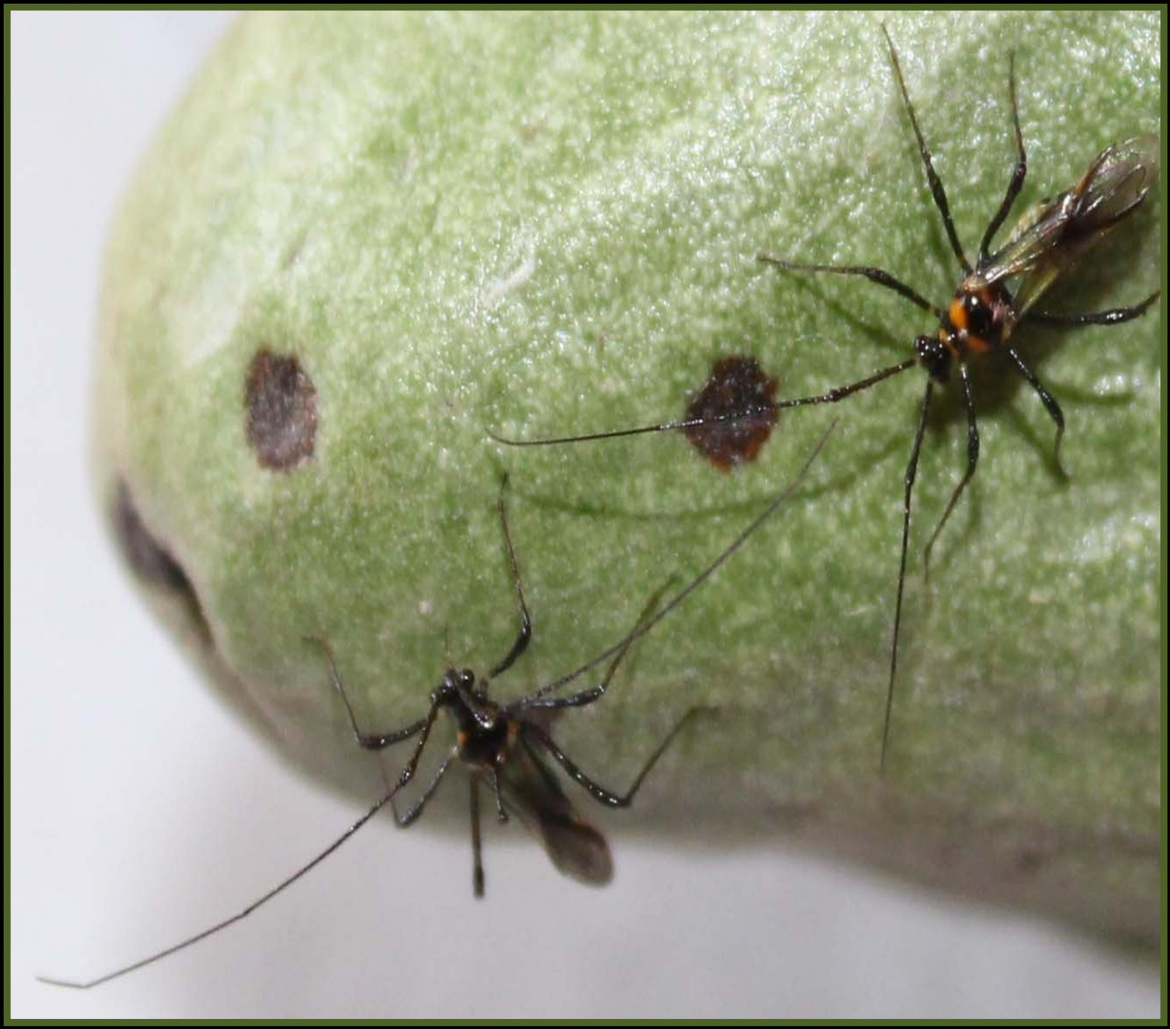
Screening on pods.

Medium matured, newly picked pods of each genotype were used for pod screening. Detached pods in insect rearing cages were subjected to artificial screening. *Helopeltis theivora* Waterhouse was released to freshly collected pods at the rate of 4 per cage (2 adult male and 2 adult female). The number of feeding lesions (Fig 2) was counted at 12 hour intervals until 72 hours, and the infestation was assessed [8].

### Scoring for TMB infestation

Based on the average number of lesions on shoots or pods, the hybrids were divided into five groups. Because the average number of lesions on shoots and pods varied greatly, a distinct grading technique was used for each, as shown in Table 2.

**Table 2:** Damage score rating on cocoa shoots and pods.

Sl. no.	Categories	No. of punctures on shoot	No. of punctures on pod
1.	Highly resistant (HR)	0–3	0–33
2.	Resistant (R)	3.01–6	33.01–66
3.	Moderately susceptible (MS)	6.01–9	66.01–99
4.	Susceptible (S)	9.01–12	99.01–132
5.	Highly susceptible (HS)	> 12.01	> 132.01

### Estimation of polyphenol in pod husk and tender shoots of cocoa

Polyphenol content was estimated by following the Folin–Ciocalteau (FC) reagent method [9]. Dewaxed cocoa pod husk and tender shoot weighing 500 mg were ground with 80percent ethanol in a mortar and pestle. This was placed in a centrifuge tube and spun for 20 minutes at 10,000 rpm. The supernatant was then poured into an evaporating dish. To collect all of the phenol present in the sample, this method was repeated for 2–3 times. Evaporating dishes were placed in a hot water bath for one hour to eliminate surplus ethanol. To the left-over residue 40 mL of distilled water was added. From that 0.2 mL aliquot was taken to a test tube and 13 mL distilled water was added followed by, addition of 0.5 mL FC reagent. The reaction mixture in the test tubes was incubated for 3 minutes. After that, a 2 mL solution of 20 percent Na2CO3 was added. These test tubes were placed in a boiling water bath for one minute before being incubated at room temperature for 60 minutes. The absorbance was measured at 650 nm using a spectrophotometer against a reagent blank. Total phenols were calculated using catechin as a reference. Concentration of phenol present in sample was calculated by substituting the absorbance value in the equation given below:

$$Total\;\mathrm{Phenol}\;(\%)=\frac{\mathrm{Optical}\;\mathrm{density}\;\mathrm{of}\;\mathrm{sample}}{\mathrm{Optical}\;\mathrm{density}\;\mathrm{of}\;\mathrm{standard}}\times \frac{\mathrm{Concentration}\;\mathrm{of}\;\mathrm{standard}}{\mathrm{Volume}\;\mathrm{of}\;\mathrm{sample}}\times 100$$

### Statistical analysis

The amount of feeding lesions on the shoot and pod, as well as total polyphenol content, were submitted to analysis of variance (ANOVA) using the WASP 2.0 package. The design used was Completely Randomized Block Design (CRD). Using SPSS software (Version 16), the relationship between total polyphenol content and the number of feeding punctures was calculated. Logistic regression analysis was also carried out to confirm whether polyphenol content can be used as a strong biochemical marker for identifying tea mosquito resistant genotypes.

## Results and discussions

The tea mosquito bug (*Helopeltis theivora* Waterhouse) is a major sucking pest that attacks young shoots, cherelles, and mature pods of cocoa. Twenty cocoa hybrids were chosen for tea mosquito insect screening in this study based on their general vigour and yield performance. The field was surrounded on three sides by old germplasm blocks and on one side by a cashew plantation. When compared to the neighboring fields, natural infestation was surprisingly low in these hybrids.

Tea mosquito bug is a polyphagous pest with a wide range of hosts including many cultivated crops like cashew, tea, guava, pepper, etc. Many weedy plants also act as alternate host [10]. In our study, since rearing on cocoa found to be a failure, culture of *Helopeltis theivora* Waterhouse was maintained on the alternate host mile-a-minute (*Mikania micrantha Kunth*) under laboratory codition in insect rearing cages. The female adults started laying eggs with in two days. Eggs are laid on leaves, tender shoots, petioles etc. The egg hatching took place within 5–10 days. Nymphs were transferred to another cage and provided with fresh feed for development. A nymph will develop into an adult within 10–12 days. Longevity and fecundity of *H*. *theivora* vary depending on rearing conditions. A mean adult longevity of 30 days was observed for *H*. *theivora* raised on cocoa pods in West Malaysia [4]. The same species was reportedto have a mean longevity of 20 days when reared on cocoa pods, but only 6 days when raised on the shoots [11].

### Screening of tea mosquito bug on budded plants and pods

Insect screening techniques differ depending on the crop and the insect in question. To achieve a consistent infestation, artificial inoculation is required. This will allow for an unbiased pest resistance screening among genotypes. To investigate the reaction of different hybrids to *Helopeltis theivora*, an artificial screening for tea mosquito insect resistance was carried out on budded plants and detached pods. By injecting its toxic saliva into the host, the tea mosquito bug feeds on new shoots and fragile sections of plants, causing the cells around the puncture site to break down. The region turned dark brown and got dried after 24 hours of feeding [12]. These dark feeding lesions are signs of tea mosquito bug attack, and the difference in size was measured in the current study and used as an indicator to screen the genotypes for tea mosquito resistance.

Table 3 depict the responses of cocoa hybrid seedlings to the tea mosquito bug. Based on the score chart, seven hybrids (PIV 59.8, PIV 60.9, PII 12.11, VSDI 33.4, PIV 56.9, VSDI 11.11 and PIV 31.9) were ranked as highly resistant to tea mosquito bug. After 72 hours of screening, all five hybrids had less than three lesions per plant. SIV 1.10, SIV 5.15, VSDI 23.21, VSDI 30.8, and PIV 26.8 hybrids were included in the resistant group, with feeding lesions ranging from 3.01 to 6 after 72 hours of screening. At the end of the screening, four hybrids showed a moderately vulnerable reactivity to tea mosquito bug attack, with 6.01 to 9 lesions. PIV 45.4, SIV 1.6, and VSDI 29.9 hybrids were classified as moderately sensitive (feeding punctures 6.01–9).Hybrids VSDI 10.13,PIV 58.6, PIII 2.3, SIV 10.11 and PIV 19.9 were under highly susceptible category since the average feeding lesions were more than 12.01 in those hybrids.

**Table 3:** Average number of feeding punctures after 72 hours of TMB release.

Hybrids	Average number of feeding punctures after 72 hours			
	On pods	Resistance reaction	On budded plants	Resistance reaction
PIV 45.4	29.00 (5.380)	HR	8.11 (2.843)	MS
PIII 2.3	22.00 (4.666)	HR	14.88 (3.857)	HS
PIV 59.8	56.30 (7.495)	R	2.66 (1.626)	HR
SIV 10.11	116.33 (10.781)	S	15.55 (3.944)	HS
VSDI 10.13	62.33 (7.890)	R	12.11 (3.474)	HS
SIV 1.10	38.33 (6.187)	R	5.11 (2.256)	R
PIV 60.9	44.66 (6.680)	R	0.96 (0.971)	HR
PII 12.11	19.10 (4.356)	HR	0.53 (0.698)	HR
SIV 5.15	173.33 (13.160)	HS	5.98 (2.444)	R
VSDI 33.4	22.40 (4.730)	HR	2.77 (1.655)	HR
VSDI 23.21	210.66 (14.513)	HS	3.44 (1.840)	R
PIV 58.6	26.00 (5.073)	HR	13.88 (3.722)	HS
PIV 56.9	13.33 (3.622)	HR	1.77 (1.323)	HR
VSDI 30.8	41.33 (6.422)	R	3.77 (1.940)	R
VSDI 11.11	102.70 (10.134)	S	0.89 (0.937)	HR
SIV 1.6	36.34 (6.020)	R	8.77 (2.961)	MS
PIV 19.9	175.70 (13.255)	HS	13.33 (3.648)	HS
PIV 26.8	122.00 (11.040)	S	4.33 (2.074)	R
PIV 31.9	11.66 (3.382)	HR	2.11 (1.445)	HR
VSDI 29.9	45.79 (6.748)	R	6.22 (2.491)	MS
**CD (0.05)**	**0.657**		**0.286**	
**CV (%)**	**15.24**		**17.51**	

*Values in parenthesis represent transformed values using angular transformation

When pod screening was carried out, seven hybrids PIV 45.4, PIII 2.3, PII 12.11, VSDI 33.4, PIV 56.9, PIV 31.9 and PIV 58.6 were found to be highly resistant to tea mosquito bug infestation with average number of feeding lesions less than 33. PIV 59.8, SIV 1.10, PIV 60.9, VSDI 30.8, SIV 1.6, VSDI 29.9, and VSDI 10.13 were classified as resistant (feeding punctures ranging from 33.01 to 66). Hybrids SIV 10.11, VSDI 11.11 and PIV 26.8 were found to be susceptible, in which feeding lesions ranged between 99.01–132. The highly susceptible hybrids were SIV 5.15, VSDI 23.21 and PIV 19.9 with number of feeding lesions more than 132.01.

### The effect of cocoa husk and shoot phenol on the resilience of tea mosquito bugs

It was observed that hybrids classified as highly resistant had significantly higher phenol content than those classified as susceptible (Table 4). The significantly low phenol content in the susceptible hybrids suggests that phenolics have a function in mediating resistance to tea mosquito bug in cocoa.

**Table 4:** Total polyphenol content in pod husk and tender shoot.

Sl. no.	Hybrids	Pod husk total polyphenol (%)	Tender shoot total polyphenol (%)
1.	PIV 45.4	0.503 (4.068)	0.543 (4.227)
2.	PIII 2.3	0.296 (3.122)	0.177 (2.411)
3.	PIV 59.8	1.259 (6.443)	0.600 (4.442)
4.	SIV 10.11	0.518 (4.096)	0.170 (2.361)
5.	VSDI 10.13	0.470 (3.930)	0.257 (2.903)
6.	SIV 1.10	0.884 (5.391)	0.247 (2.849)
7.	PIV 60.9	1.440 (6.896)	0.256 (2.902)
8.	PII 12.11	3.670 (11.043)	0.133 (2.087)
9.	SIV 5.15	0.427 (3.745)	0.520 (4.137)
10.	VSDI 33.4	1.563 (7.183)	0.387 (3.565)
11.	VSDI 23.21	0.051 (1.277)	0.539 (4.210)
12.	PIV 58.6	0.163 (2.315)	0.192 (2.506)
13.	PIV 56.9	1.518 (7.075)	0.477 (3.959)
14.	VSDI 30.8	1.041 (5.844)	0.254 (2.891)
15.	VSDI 11.11	0.427 (3.744)	0.590 (4.405)
16.	SIV 1.6	1.385 (6.763)	0.244 (2.833)
17.	PIV 19.9	0.697 (4.788)	0.137 (2.118)
18.	PIV 26.8	0.280 (3.031)	0.413 (3.685)
19.	PIV 31.9	0.670 (4.694)	0.385 (3.557)
20.	VSDI 29.9	0.657 (4.646)	0.415 (3.692)
**CD (0.05)**		**0.423**	**0.083**
**CV (%)**		**5.119**	**3.524**

* Figures in parenthesis are transformed values using angular transformation

**Fig 3:**
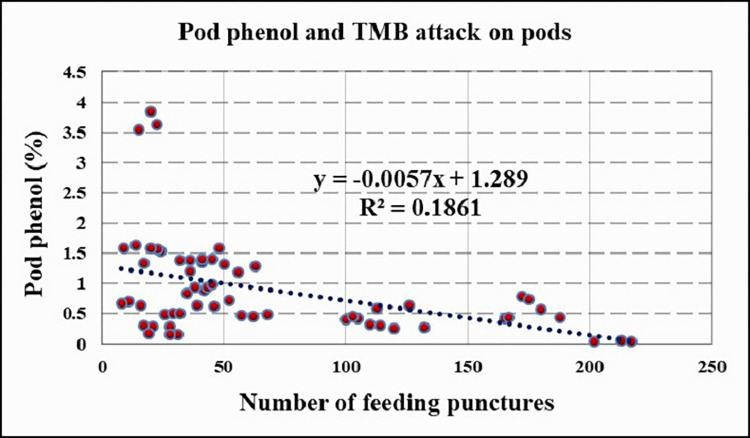
Correlation between pod phenol (%) and number of feeding lesion on pod.

**Fig 4:**
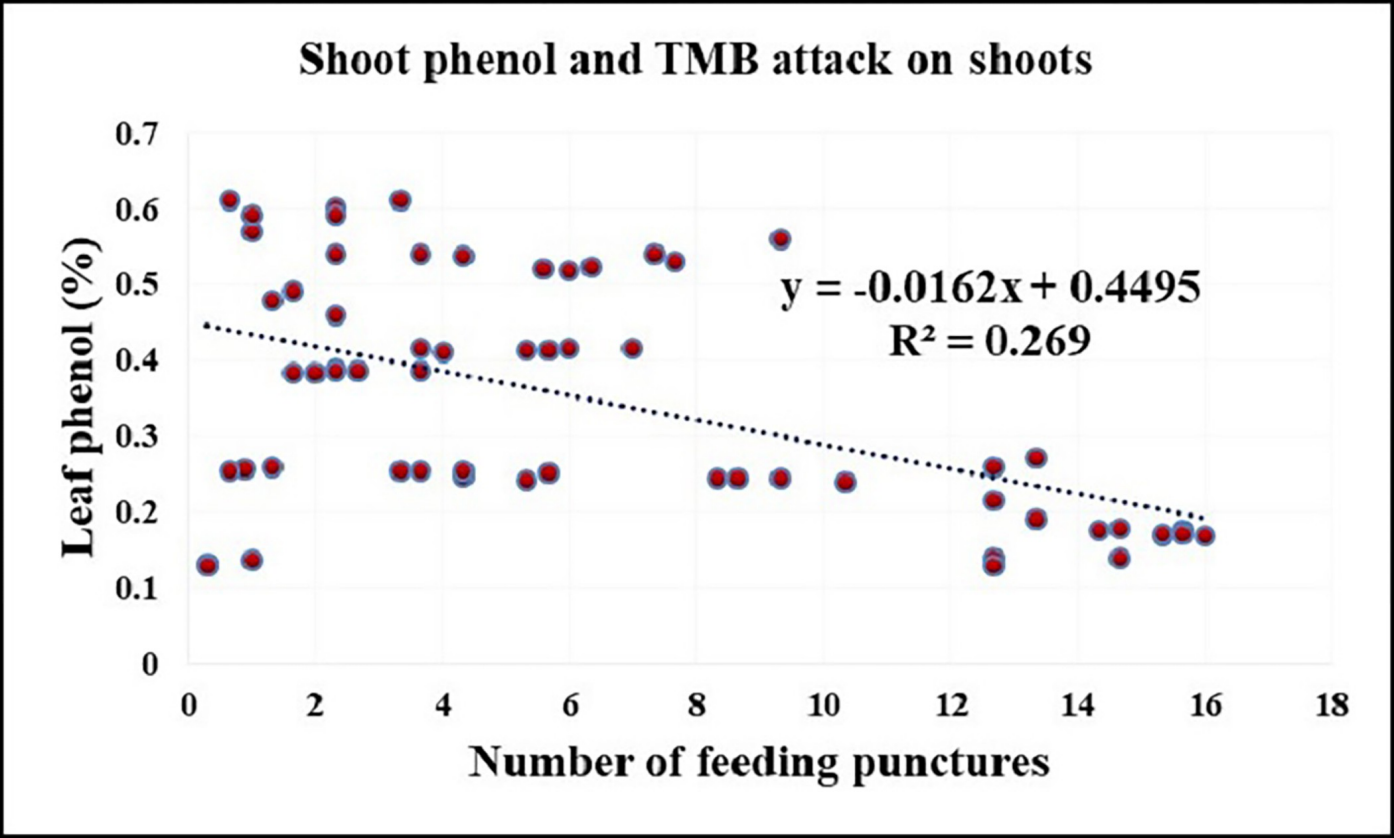
Correlation between shoot phenol (%) and number of feeding lesion on shoot.

Pearson’s correlation analysis was carried out to determine if there was a link between polyphenol content and tea mosquito bug attack, and it was discovered that total phenol content in both pod husk and shoot was inversely related to TMB attack, with a moderate and high significant negative correlation of 0.431 and 0.518, respectively (Figs 3 and 4).

Plants use phenolic heteropolymers to defend themselves against insects and diseases [13]. They usually bind to protein, decreasing the availability of food protein for insects or blocking enzyme activity [14]. Certain varieties of tea were found to be more resistant to TMB because they maintain larger amounts of phenolics in the face of attack [15]. In the case of cashew, sensitive genotypes have lower phenol content than tolerant genotypes [16–18].

Regression analysis was conducted to confirm the influence of polyphenol towards tea mosquito bug resistance (Table 5). Based on Exp(B) value from the regression model, expected percentage of improvement for tea mosquito bug resistance over the base population was calculated and it was found that if selection is based on phenol content, new population formed from the base population will express 84.098 per cent of improvement regarding the resistance. From correlation and regression analysis it is confirmed that phenol content can be used as a potential marker indicating the level of resistance of cocoa hybrids against tea mosquito bug resistance. Phenolic compounds present in plants are having role in plant defense response against biotic stresses [19, 20] and it was confirmed in different crops including rice, apple, cucumber *etc*.

**Table 5:** Logistic estimate of biochemical constituent influencing tea mosquito bug resistance.

Variable	Coefficient	Standard error	Wald	Significance	Exp (B)	Expected per cent of improvement over Population
**Phenol** **	2.485	1.942	1.636	0.201	11.998	84.098
Constant	-0.327	0.671	0.238	0.625	0.721	

**Significance value less than 0.625

## Conclusion

The study clearly indicated that polyphenol content in cocoa has a great influence on conferring resistance against tea mosquito bug in cocoa and can serve as a selection criteria for identifying cocoa genotypes with tea mosquito bug resistance. Even though this is a preliminary study, it represents a good step toward understanding the tea mosquito bug resistance mechanism in cocoa and will be a foundation for future researches on this topic.

## Supporting information

S1 Table Correlation studies between shoot characters and TMB attack. (DOCX)

S2 Table Correlation between pod morphological characters and TMB attack. (DOCX)
