# Supplementary material for: Deciphering the role of polyphenol in defence mechanism against tea mosquito bug (Helopeltis theivora Waterhouse.) in cocoa (Theobroma cocoa L.)
Source: PLoS One. 2022 Oct 14;17(10):e0271432. doi: 10.1371/journal.pone.0271432 (PMC9565741; doi:10.1371/journal.pone.0271432)
Supplement: S1 Table — (DOCX) [file pone.0271432.s001.docx]

|  |  | | | | | |
| --- | --- | --- | --- | --- | --- | --- |
|  | | Shoot Phenol | TMB Attack | Shoot Colour | Shoot Texture |  |
| Shoot Phenol | | 1 |  |  |  |  |
| TMB Attack | | - 0.518 | 1 |  |  |  |
| Shoot Colour | | 0.282 | -0.359 | 1 |  |  |
| Shoot Texture | | 0.070 | -0.406 | 0.206 | 1 |  |

**Table S1. Correlation studies between shoot characters and TMB attack**
