# Supplementary material for: Deciphering the role of polyphenol in defence mechanism against tea mosquito bug (Helopeltis theivora Waterhouse.) in cocoa (Theobroma cocoa L.)
Source: PLoS One. 2022 Oct 14;17(10):e0271432. doi: 10.1371/journal.pone.0271432 (PMC9565741; doi:10.1371/journal.pone.0271432)
Supplement: S2 Table — (DOCX) [file pone.0271432.s002.docx]

|  | TMB attack | Shape | Colour | Apex | Base | Rugosity |
| --- | --- | --- | --- | --- | --- | --- |
| TMB attack | 1 |  |  |  |  |  |
| Shape | -0.141 | 1 |  |  |  |  |
| Colour | -0.086 | .000 | 1 |  |  |  |
| Apex | -0.265 | 0.601^**^ | -0.031 | 1 |  |  |
| Base | -0.081 | -0.711^**^ | 0.040 | -0.282 | 1 |  |
| Rugosity | -0.045 | -0.313 | 0.362 | -0.232 | 0.381 | 1 |

| **. Correlation is significant at the 0.01 level (2-tailed). | |
| --- | --- |
|  |  |

**Table S2. Correlation between pod morphological characters and TMB attack**
