# Supplementary material for: Deciphering the role of polyphenol in defence mechanism against tea mosquito bug (Helopeltis theivora Waterhouse.) in cocoa (Theobroma cocoa L.)
Source: PLoS One. 2022 Oct 14;17(10):e0271432. doi: 10.1371/journal.pone.0271432 (PMC9565741; doi:10.1371/journal.pone.0271432)
Supplement: Attachment — Submitted filename: PONE-D-22-07611.pdf [file pone.0271432.s003.pdf]

PLOS ONE

Deciphering the role of polyphenol in defence mechanism against Tea mosquito bug (Helopeltis theivora Waterhouse.) in cocoa (Theobroma cocoa L.)

--Manuscript Draft--

**Manuscript Number:**	PONE-D-22-07611
**Article Type:**	Research Article
**Full Title:**	Deciphering the role of polyphenol in defence mechanism against Tea mosquito bug (Helopeltis theivora Waterhouse.) in cocoa (Theobroma cocoa L.)
**Short Title:**	Tea mosquito bug resistance in cocoa
**Corresponding Author:**	Minimol JS<br>Kerala Agricultural University<br>Thrissur, Kerala INDIA
**Keywords:**	Cocoa; tea mosquito bug; resistance breeding; phenol
**Abstract:**	Tea mosquito bug (TMB) is a serious pest of cocoa whose prevalence is high, mostly during summer and post monsoon season. Three species of tea mosquito have been reported on cocoa: Helopeltis antonii , H. theivora , and H. bradyi . H. theivora is the most prevalent one causing damage to young shoots, cherelles and pods. Rearing of tea mosquito on cocoa was found to be a failure in the present study hence Helopeltis theivora Waterhouse was maintained on the alternate host mile-a-minute (Mikania micrantha) under laboratory condition in insect rearing cages. Using freshly reared bugs twenty cocoa hybrids were screened for resistance and PIV 59.8, PIV 60.9, PII 12.11, VSDI 33.4, and PIV 56.9 were ranked as highly resistant after 72 hours of screening. All these five hybrids had less than three lesions per plant. It was observed that hybrids classified as highly resistant had significantly higher phenol content than those classified as susceptible. The significantly low phenol content in the susceptible hybrids suggests that phenolics have a function in mediating resistance to tea mosquito bug in cocoa. From correlation and regression analysis it is confirmed that phenol content can be used as a potential marker indicating the level of resistance of cocoa hybrids against tea mosquito bug resistance. Resistant hybrids served as parents in polyclonal garden for production of hybrid seedlings.
**Order of Authors:**	Shilpa K. S.
	Minimol JS
	Gavas Rakesh
	B. Suma
	Jiji Joseph
	Maheswarappa H.P.
	P.S. Panjami
**Additional Information:**	
**Question**	**Response**
**Financial Disclosure**<br><br>Enter a financial disclosure statement that describes the sources of funding for the work included in this submission. Review the submission guidelines for detailed requirements. View published research articles from *PLOS ONE* for specific examples.	Project was funded by Kerala Agricultural University as a part of student project and also supported by Mondelez India Foods Ltd

Powered by Editorial Manager® and ProduXion Manager® from Aries Systems Corporation

This statement is required for submission and **will appear in the published article** if the submission is accepted. Please make sure it is accurate.<br><br>**Unfunded studies**<br>Enter: *The author(s) received no specific funding for this work.*<br><br>**Funded studies**<br>Enter a statement with the following details:<br>• Initials of the authors who received each award<br>• Grant numbers awarded to each author<br>• The full name of each funder<br>• URL of each funder website<br>• Did the sponsors or funders play any role in the study design, data collection and analysis, decision to publish, or preparation of the manuscript?<br>• **NO** - Include this sentence at the end of your statement: *The funders had no role in study design, data collection and analysis, decision to publish, or preparation of the manuscript.*<br>• **YES** - Specify the role(s) played.<br><br>* typeset	
**Competing Interests**<br><br>Use the instructions below to enter a competing interest statement for this submission. On behalf of all authors, disclose any competing interests that could be perceived to bias this work—acknowledging all financial support and any other relevant financial or non-financial competing interests.<br><br>This statement is required for submission and **will appear in the published article** if the submission is accepted. Please make sure it is accurate and that any funding sources listed in your Funding Information later in the submission form are also declared in your Financial Disclosure statement.<br><br>View published research articles from *PLOS ONE* for specific examples.	The authors have declared that no competing interests exist

Powered by Editorial Manager® and ProduXion Manager® from Aries Systems Corporation

**NO authors have competing interests**<br><br>Enter: *The authors have declared that no competing interests exist.*<br><br>**Authors with competing interests**<br><br>Enter competing interest details beginning with this statement:<br><br>*I have read the journal's policy and the authors of this manuscript have the following competing interests: [insert competing interests here]*<br><br>* typeset	
**Ethics Statement**<br><br>Enter an ethics statement for this submission. This statement is required if the study involved:<br><br>• Human participants<br>• Human specimens or tissue<br>• Vertebrate animals or cephalopods<br>• Vertebrate embryos or tissues<br>• Field research<br><br>Write "N/A" if the submission does not require an ethics statement.<br><br>General guidance is provided below. Consult the submission guidelines for detailed instructions. **Make sure that all information entered here is included in the Methods section of the manuscript.**	NA

Powered by Editorial Manager® and ProduXion Manager® from Aries Systems Corporation

**Format for specific study types**<br><br>**Human Subject Research (involving human participants and/or tissue)**<br>• Give the name of the institutional review board or ethics committee that approved the study<br>• Include the approval number and/or a statement indicating approval of this research<br>• Indicate the form of consent obtained (written/oral) or the reason that consent was not obtained (e.g. the data were analyzed anonymously)<br><br>**Animal Research (involving vertebrate animals, embryos or tissues)**<br>• Provide the name of the Institutional Animal Care and Use Committee (IACUC) or other relevant ethics board that reviewed the study protocol, and indicate whether they approved this research or granted a formal waiver of ethical approval<br>• Include an approval number if one was obtained<br>• If the study involved *non-human primates*, add *additional details* about animal welfare and steps taken to ameliorate suffering<br>• If anesthesia, euthanasia, or any kind of animal sacrifice is part of the study, include briefly which substances and/or methods were applied<br><br>**Field Research**<br><br>Include the following details if this study involves the collection of plant, animal, or other materials from a natural setting:<br>• Field permit number<br>• Name of the institution or relevant body that granted permission	
**Data Availability**<br><br>Authors are required to make all data underlying the findings described fully available, without restriction, and from the time of publication. PLOS allows rare exceptions to address legal and ethical concerns. See the PLOS Data Policy and FAQ for detailed information.	Yes - all data are fully available without restriction

Powered by Editorial Manager® and ProduXion Manager® from Aries Systems Corporation

A Data Availability Statement describing where the data can be found is required at submission. Your answers to this question constitute the Data Availability Statement and **will be published in the article**, if accepted.<br><br>**Important:** Stating 'data available on request from the author' is not sufficient. If your data are only available upon request, select 'No' for the first question and explain your exceptional situation in the text box.<br><br>Do the authors confirm that all data underlying the findings described in their manuscript are fully available without restriction?	
**Describe where the data may be found in full sentences. If you are copying our sample text, replace any instances of XXX with the appropriate details.**<br><br>• If the data are **held or will be held in a public repository**, include URLs, accession numbers or DOIs. If this information will only be available after acceptance, indicate this by ticking the box below. For example: *All XXX files are available from the XXX database (accession number(s) XXX, XXX.*).<br>• If the data are all contained **within the manuscript and/or Supporting Information files**, enter the following: *All relevant data are within the manuscript and its Supporting Information files.*<br>• If neither of these applies but you are able to provide **details of access elsewhere**, with or without limitations, please do so. For example:<br><br>*Data cannot be shared publicly because of [XXX]. Data are available from the XXX Institutional Data Access / Ethics Committee (contact via XXX) for researchers who meet the criteria for access to confidential data.*<br><br>*The data underlying the results presented in the study are available from (include the name of the third party*	NA

Powered by Editorial Manager® and ProduXion Manager® from Aries Systems Corporation

and contact information or URL).<br>• This text is appropriate if the data are owned by a third party and authors do not have permission to share the data.<br><br>* typeset	
Additional data availability information:	Tick here if the URLs/accession numbers/DOIs will be available only after acceptance of the manuscript for publication so that we can ensure their inclusion before publication.

Powered by Editorial Manager® and ProduXion Manager® from Aries Systems Corporation

Manuscript
Click here to access/download;Manuscript;Manuscript.pdf

Deciphering the role of polyphenol in defence mechanism against Tea mosquito bug (Helopeltis theivora Waterhouse.) in cocoa (Theobroma cocoa L.)

Shilpa K. S.[1¶], Minimol J. S.[2 ¶*], Gavas Rakesh[3¶], Suma B.[4&], Jiji Joseph[5&], Maheswarappa H. P.[6&], Panjami P.S.7[&]

[1]MSc. Student, Department of Plant Breeding and Genetics, College of Agriculture, Kerala Agricultural University, Vellanikkara, Kerala, India

[2]Associate Professor, Plant Breeding and Genetics, Cocoa Research Centre, Kerala Agricultural University, Vellanikkara, Kerala, India

[3]Assistant Professor, Agricultural Entomology, Banana Research Station, Kerala Agricultural University, Vellanikkara, Kerala, India

[4]Professor and Head, Cocoa Research Centre, Kerala Agricultural University, Vellanikkara, Kerala, India

[5]Professor and Head, Department of Plant Breeding and Genetics, College of Agriculture, Kerala Agricultural University, Vellanikkara, Kerala, India

[6]Director of Research, University of Horticulture Sciences, Bagalkot, India

[7]Assistant Professor, Kerala Agricultural University, Vellanikkara, Kerala, India

* Corresponding Author

E-mail: minimol.js@kau.in

[¶]These authors contributed equally to this work.

[&]These authors also contributed equally to this work.

Abstract

Tea mosquito bug (TMB) is a serious pest of cocoa whose prevalence is high, mostly during summer and post monsoon season. Three species of tea mosquito have been reported on cocoa: *Helopeltis antonii*, *H. theivora*, and *H. bradyi*. *H. theivora* is the most prevalent one causing damage to young shoots, cherelles and pods. Rearing of tea mosquito on cocoa was found to be a failure in the present study hence *Helopeltis theivora* Waterhouse was maintained on the alternate host mile-a-minute (*Mikania micrantha*) under laboratory condition in insect rearing cages. Using freshly reared bugs twenty cocoa hybrids were screened for resistance and PIV 59.8, PIV 60.9, PII 12.11, VSDI 33.4, and PIV 56.9 were ranked as highly resistant after 72 hours of screening. All these five hybrids had less than three lesions per plant. It was observed that hybrids classified as highly resistant had significantly higher phenol content than those classified as susceptible. The significantly low phenol content in the susceptible hybrids suggests that phenolics have a function in mediating resistance to tea mosquito bug in cocoa. From correlation and regression analysis it is confirmed that phenol content can be used as a potential marker indicating the level of resistance of cocoa hybrids against tea mosquito bug resistance. Resistant hybrids served as parents in polyclonal garden for production of hybrid seedlings.

Keywords: cocoa, tea mosquito bug, polyphenol, correlation, regression

Introduction

Insects belonging to the family Miridae are serious pests of cocoa (*Theobroma cacao* L.) worldwide [1]. Tea mosquito bug (TMB), is a significant mirid pest of cocoa, whose prevalence has increased seriously during the most recent couple of years in summer and post monsoon season. Three species of tea mosquito have been reported on cocoa: *Helopeltis antonii, H. theivora*, and *H. bradyi*. *H. theivora* is the most prevalent one causing damage to young shoots,

cherelles and pods [2]. Feeding lesions produced by tea mosquito bug can kill small cherelle, while older pods can continue to grow even if badly injured [3]. However, the yield is harmed as a result of malformed pods [4]. The cocoa pollinator (*Forcipomyia* spp.) and the pest both are the members of the Miridae family. As a result, chemical control becomes more complicated. Insecticides used to reduce tea mosquitos also limit the pollinator population, resulting in considerable crop losses. The development and deployment of tea mosquito bug resistant cocoa genotypes is the greatest alternative to chemical control [5]. Identification of a morphological marker is a crucial step in confirming resistance. Plant polyphenols are secondary metabolites, and they are one of the most abundant compounds in plants. Polyphenols are thought to have a key function in imparting resistance to many insect pests [6]. Hence this investigation was carried out with the goal of identifying tea mosquito-resistant cocoa genotypes and determining the effect of polyphenol in imparting resistance.

Materials and methods

Twenty cocoa hybrids were evaluated for tea mosquito resistance in Cocoa Research Centre, Kerala Agricultural University. Details of hybrids are presented in Table 1.

Table 1. List of hybrids selected for tea mosquito bug screening

Sl. no.	Hybrids	Parentage
1.	PIV 45.4	GI5.9 x GI10.2
2.	PIII 2.3	H7.1 x H5.3
3.	PIV 59.8	H10.1 x H6.8
4.	SIV 10.11	GI5.9 x GI10.2
5.	VSDI 10.13	GIV126 x GIV18.5
6.	SIV 1.10	GIV$_{68}$ x GI$_{5.9}$
7.	PIV 60.9	GII20.4 x GI5.9
8.	PII 12.11	GIV$_{24}$ x GIV$_{51}$
9.	SIV 5.15	GII20.4 x GI5.9
10.	VSDI 33.4	GIV148 x GIV18.5

11.	VSDI 23.21	GIV171 x GIV18.5
12.	PIV 58.6	GII20.4 x GI5.9
13.	PIV 56.9	GIV148 x GIV18.5
14.	VSDI 30.8	GIV18.8 x GIV18.5
15.	VSDI 11.11	GVI126 x GIV18.5
16.	SIV 1.6	GVI$_{51}$ x GI$_{5.9}$
17.	PIV 19.9	H5.3 x H6.1
18.	PIV 26.8	H7.10 x H3.5
19.	PIII 15.9	H10.1 x H6.8
20.	VSDI 29.9	GVI$_{188}$ x GVI$_{55}$

Collection and rearing of TMB (*Helopeltis theivora* Waterhouse.)

The initial culture of tea mosquito bug was established with collections from the farm of Cocoa Research Centre. Adult male and females were collected from the field during morning hours. Attempt to rare it on cocoa seedlings was a failure due to lack of oviposition in cocoa. The alternate host mile-a-minute (*Mikania micrantha*) was utilised to combat the problem. [7] developed a standard procedure for this purpose, which was followed. Newly reared insects collected from the raring cage were used for further screening procedure.

Screening of tea mosquito bug on cocoa seedlings

Patch budding was done on six-month-old root stock to raise the screening materials. Budded plants were brought up in the nursery. Screening was done on six-month-old budded plant. Three replications having five budded plants of each hybrid were screened inside the insect net house facility. Freshly reared *Helopeltis theivora* were introduced into the insect net house at a rate of 100 adults (50 male and 50 female) for every screening test. The severity of the infestation on the shoots/leaves was measured by counting the number of feeding lesions for every 12 hours interval for 72 hours and then scoring it (Srikumar and Bhat, 2013) [8].

Fig 1. Screening on budded plants

Screening of tea mosquito bug on pods

Medium matured, newly picked pods of each genotype were used for pod screening. Detached pods in insect rearing cages were subjected to artificial screening. *Helopeltis theivora* Waterhouse was released to freshly collected pods at the rate of 4 per cage (2 adult male and 2 adult female). The number of feeding lesions was counted at 12 hour intervals until 72 hours, and the infestation was assessed [8] (Fig 2).

Fig 2. Screening on pods

Scoring for TMB infestation

Based on the average number of lesions on shoots or pods, the hybrids were divided into five groups. Because the average number of lesions on shoots and pods varied greatly, a distinct grading technique was used for each, as shown in Table 2.

Table 2. Damage score rating on cocoa shoots and pods

Sl. no.	Categories	No. of punctures on shoot	No. of punctures on pod
1.	Highly resistant (HR)	0 – 3	0 – 33
2.	Resistant (R)	3.01 – 6	33.01 – 66
3.	Moderately susceptible (MS)	6.01 – 9	66.01 – 99
4.	Susceptible (S)	9.01 – 12	99.01 – 132
5.	Highly susceptible (HS)	> 12.01	> 132.01

Estimation of polyphenol in pod husk and tender shoots of cocoa

Polyphenol content was estimated by following the Folin – Ciocalteau (FC) reagent method given by [9]. Dewaxed cocoa pod husk and tender shoot weighing 500 mg were ground with 80percent ethanol in a mortar and pestle. This was placed in a centrifuge tube and spun for 20 minutes at 10,000 rpm. The supernatant was then poured into an evaporating dish. To collect all of the phenol present in the sample, this method was repeated for 2-3 times. Evaporating dishes were placed in a hot water bath for one hour to eliminate surplus ethanol.

Evaporating dishes were placed in a hot water bath for one hour to eliminate surplus ethanol. To the left-over residue 40 mL of distilled water was added. From that 0.2 mL aliquot was taken to a test tube and 13 mL distilled water was added followed by, addition of 0.5 mL FC reagent. The reaction mixture in the test tubes was incubated for 3 minutes. After that, a 2 mL solution of 20 percent Na2CO3 was added. These test tubes were placed in a boiling water bath for one minute before being incubated at room temperature for 60 minutes. The absorbance was measured at 650 nm using a spectrophotometer against a reagent blank. Total phenols were calculated using catechin as a reference. Concentration of phenol present in sample was calculated by substituting the absorbance value in the equation given below:

$$\text{Total Phenol (\%)} = \frac{\text{Optical density of sample}}{\text{Optical density of standard}} \times \frac{\text{Concentration of standard}}{\text{Volume of sample}} \times 100$$

Statistical analysis

The amount of feeding lesions on the shoot and pod, as well as total polyphenol content, were submitted to analysis of variance (ANOVA) using the WASP 2.0 package. The design used was Completely Randomized Block Design (CRD). Using SPSS software (Version 16), the relationship between total polyphenol content and the number of feeding punctures was calculated. Logistic regression analysis was also carried out to confirm whether polyphenol content can be used as a strong biochemical marker for identifying tea mosquito resistant genotypes.

Results and discussions

The tea mosquito bug (*Helopeltis theivora* Waterhouse) is a major sucking pest that attacks young shoots, cherelles, and mature pods of cocoa. Twenty cocoa hybrids were chosen for tea mosquito insect screening in this study based on their general vigour and yield performance. The field was surrounded on three sides by old germplasm blocks and on one side by a cashew

plantation. When compared to the neighboring fields, natural infestation was surprisingly low in these hybrids.

Tea mosquito bug is a polyphagous pest with a wide range of hosts including many cultivated crops like cashew, tea, guava, pepper, etc. Many weedy plants also act as alternate host [10]. In our study, since rearing on cocoa found to be a failure, culture of *Helopeltis theivora* Waterhouse was maintained on the alternate host mile-a-minute (*Mikania micrantha*) under laboratory condition in insect rearing cages. The female adults started laying eggs with in two days. Eggs are laid on leaves, tender shoots, petioles etc. The egg hatching took place within 5-10 days. Nymphs were transferred to another cage and provided with fresh feed for development. A nymph will develop into an adult within 10-12 days. Longevity and fecundity of *H. theivora* vary depending on rearing conditions. [4] recorded a mean adult longevity of 30 days for *H. theivora* raised on cocoa pods in West Malaysia. The same species was reported by [11] to have a mean longevity of 20 days when reared on cocoa pods, but only 6 days when raised on the shoots.

Screening of tea mosquito bug on budded plants and pods

Insect screening techniques differ depending on the crop and the insect in question. To achieve a consistent infestation, artificial inoculation is required. This will allow for an unbiased pest resistance screening among genotypes. To investigate the reaction of different hybrids to *Helopeltis theivora*, an artificial screening for tea mosquito insect resistance was carried out on budded plants and detached pods. By injecting its toxic saliva into the host, the tea mosquito bug feeds on new shoots and fragile sections of plants, causing the cells around the puncture site to break down. The region turned dark brown and got dried after 24 hours of feeding [12]. These dark feeding lesions are signs of tea mosquito bug attack, and the difference in size was measured in the current study and used as an indicator to screen the genotypes for tea mosquito resistance (Plate 1).

Table 3 depict the responses of cocoa hybrid seedlings to the tea mosquito bug. Based on the score chart [8], five hybrids (PIV 59.8, PIV 60.9, PII 12.11, VSDI 33.4, and PIV 56.9) were ranked as highly resistant to tea mosquito bug. After 72 hours of screening, all five hybrids had less than three lesions per plant. SIV 5.15, VSDI 23.21, VSDI 11.11, PIV 31.9, and VSDI 29.9 hybrids were included in the resistant group, with feeding lesions ranging from 3.01 to 6 after 72 hours of screening. At the end of the screening, four hybrids showed a moderately vulnerable reactivity to tea mosquito bug attack, with 6.01 to 9 lesions. SIV 1.10, VSDI 30.8, SIV 1.6, and PIV 26.8 hybrids were classified as moderately sensitive (feeding punctures 6.01–9).Hybrid PIV 45.4, VSDI 10.13 and PIV 58.6 were grouped as susceptible and hybrids PIII 2.3, SIV 10.11 and PIV 19.9 were under highly susceptible category since the average feeding lesions were more than 12.01 in those hybrids.

Table 3. Average number of feeding punctures after 72 hours of TMB release

**Sl. no.**	**Hybrids**	**Average number of feeding punctures after 72 hours**	
		**Pods**	**Budded plants**
1.	PIV 45.4	29.00 (5.380)	8.11 (2.843)
2.	PIII 2.3	22.00 (4.666)	14.88 (3.857)
3.	PIV 59.8	56.30 (7.495)	2.66 (1.626)
4.	SIV 10.11	116.33 (10.781)	15.55 (3.944)
5.	VSDI 10.13	62.33 (7.890)	12.11 (3.474)
6.	SIV 1.10	38.33 (6.187)	5.11 (2.256)
7.	PIV 60.9	44.66 (6.680)	0.96 (0.971)
8.	PII 12.11	19.10 (4.356)	0.53 (0.698)
9.	SIV 5.15	173.33 (13.160)	5.98 (2.444)
10.	VSDI 33.4	22.40 (4.730)	2.77 (1.655)

11.	VSDI 23.21	210.66 (14.513)	3.44 (1.840)
12.	PIV 58.6	26.00 (5.073)	13.88 (3.722)
13.	PIV 56.9	13.33 (3.622)	1.77 (1.323)
14.	VSDI 30.8	41.33 (6.422)	3.77 (1.940)
15.	VSDI 11.11	102.70 (10.134)	0.89 (0.937)
16.	SIV 1.6	36.34 (6.020)	8.77 (2.961)
17.	PIV 19.9	175.70 (13.255)	13.33 (3.648)
18.	PIV 26.8	122.00 (11.040)	4.33 (2.074)
19.	PIV 31.9	11.66 (3.382)	2.11 (1.445)
20.	VSDI 29.9	45.79 (6.748)	6.22 (2.491)
**CD (0.05)**		**0.657**	**0.286**
**CV (%)**		**15.24**	**17.51**

*Values in parenthesis represent transformed values

When pod screening was carried out, seven hybrids PIV 45.4, PIII 2.3, PII 12.11, VSDI 33.4, PIV 56.9, PIV 31.9 and PIV 58.6 were found to be highly resistant to tea mosquito bug infestation with average number of feeding lesions less than 33. PIV 59.8, SIV 1.10, PIV 60.9, VSDI 30.8, SIV 1.6, VSDI 29.9, and VSDI 10.13 were classified as resistant (feeding punctures ranging from 33.01 to 66). Hybrids SIV 10.11, VSDI 11.11 and PIV 26.8 were found to be susceptible, in which feeding lesions ranged between 99.01 – 132. The highly susceptible hybrids were SIV 5.15, VSDI 23.21 and PIV 19.9 with number of feeding lesions more than 132.01.

The effect of cocoa husk and shoot phenol on the resilience of tea mosquito bugs

It was observed that hybrids classified as highly resistant had significantly higher phenol content than those classified as susceptible (Table 4). The significantly low phenol content in the susceptible hybrids suggests that phenolics have a function in mediating resistance to tea mosquito bug in cocoa.

Table 4. Total polyphenol content in pod husk and tender shoot

Sl. no.	Hybrids	Pod husk total polyphenol (%)	Tender shoot total polyphenol (%)
1.	PIV 45.4	0.503 (4.068)	0.543 (4.227)
2.	PIII 2.3	0.296 (3.122)	0.177 (2.411)
3.	PIV 59.8	1.259 (6.443)	0.600 (4.442)
4.	SIV 10.11	0.518 (4.096)	0.170 (2.361)
5.	VSDI 10.13	0.470 (3.930)	0.257 (2.903)
6.	SIV 1.10	0.884 (5.391)	0.247 (2.849)
7.	PIV 60.9	1.440 (6.896)	0.256 (2.902)
8.	PII 12.11	3.670 (11.043)	0.133 (2.087)
9.	SIV 5.15	0.427 (3.745)	0.520 (4.137)
10.	VSDI 33.4	1.563 (7.183)	0.387 (3.565)
11.	VSDI 23.21	0.051 (1.277)	0.539 (4.210)
12.	PIV 58.6	0.163 (2.315)	0.192 (2.506)
13.	PIV 56.9	1.518 (7.075)	0.477 (3.959)
14.	VSDI 30.8	1.041 (5.844)	0.254 (2.891)
15.	VSDI 11.11	0.427 (3.744)	0.590 (4.405)
16.	SIV 1.6	1.385 (6.763)	0.244 (2.833)
17.	PIV 19.9	0.697 (4.788)	0.137 (2.118)
18.	PIV 26.8	0.280 (3.031)	0.413 (3.685)
19.	PIV 31.9	0.670 (4.694)	0.385 (3.557)
20.	VSDI 29.9	0.657 (4.646)	0.415 (3.692)
**CD (0.05)**		**0.423**	**0.083**
**CV (%)**		**5.119**	**3.524**

* Figures in parenthesis are transformed values using angular transformation

Pearson's correlation analysis was carried out to determine if there was a link between polyphenol content and tea mosquito bug attack, and it was discovered that total phenol content in both pod husk and shoot was inversely related to TMB attack, with a high significant negative correlation of 0.431 and 0.518, respectively (Fig. 1 and Fig. 2).

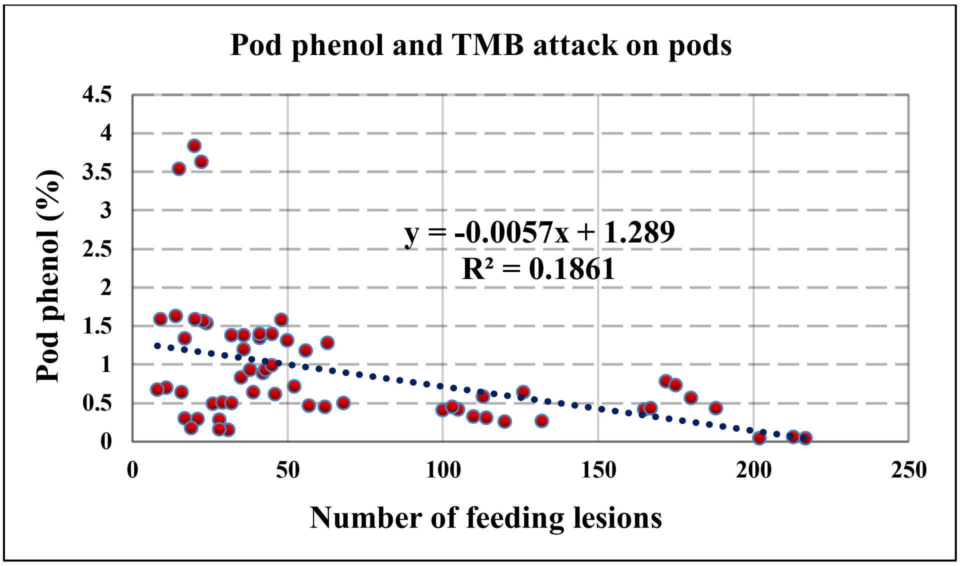

Figure 1. Correlation between pod phenol (%) and number of feeding lesions on pod

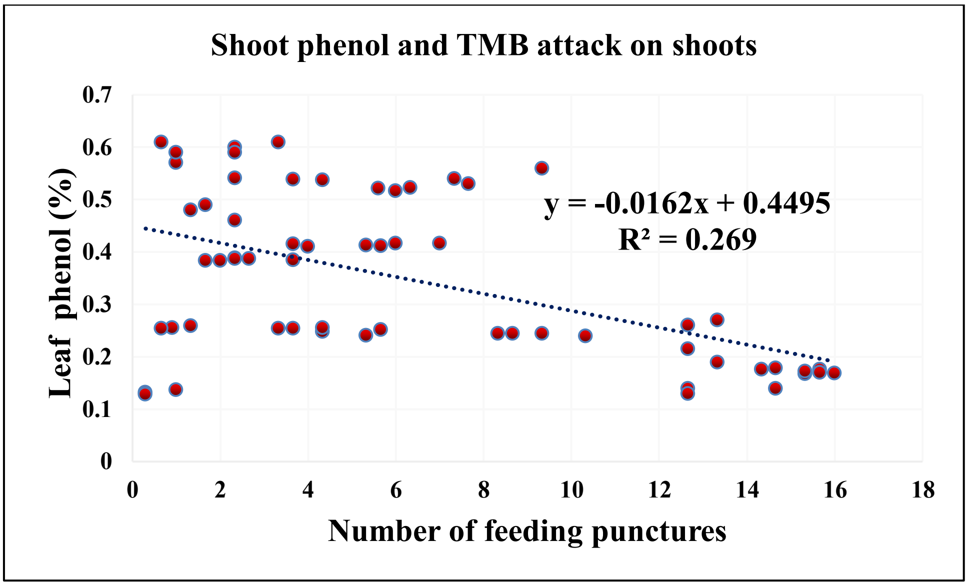

Figure 2. Correlation between leaf phenol (%) and number of feeding lesions on shoot

Plants use phenolic heteropolymers to defend themselves against insects and diseases [13]. They usually bind to protein, decreasing the availability of food protein for insects or blocking enzyme activity [14]. According to [15], certain varieties of tea are more resistant to TMB

because they maintain larger amounts of phenolics in the face of attack. In the case of cashew, sensitive genotypes have lower phenol content than tolerant genotypes [16, 17, 18].

Regression analysis was conducted to confirm the influence of polyphenol towards tea mosquito bug resistance (Table 5). Based on Exp(B) value from the regression model, expected percentage of improvement for tea mosquito bug resistance over the base population was calculated and it was found that if selection is based on phenol content, new population formed from the base population will express 84.098 per cent of improvement regarding the resistance. From correlation and regression analysis it is confirmed that phenol content can be used as a potential marker indicating the level of resistance of cocoa hybrids against tea mosquito bug resistance. Phenolic compounds present in plants are having role in plant defense reponse against biotic stresses [19, 20] and it was confirmed in different crops including rice, apple, cucumber *etc*.

Table 5 Logistic estimate of biochemical constituent influencing tea mosquito bug resistance

Variable	Coefficient	Standard error	Wald	Significance	Exp (B)	Expected per cent of improvement over Population
**Phenol****	2.485	1.942	1.636	0.201	11.998	84.098
Constant	-0.327	0.671	0.238	0.625	0.721	
**Significance value less than 0.625						

Conclusion

The study clearly indicated that polyphenol content in cocoa has a great influence on conferring resistance against tea mosquito bug in cocoa and can serve as a selection criteria for identifying cocoa genotypes with tea mosquito bug resistance. Even though this is a preliminary study, it represents a good step toward understanding the tea mosquito bug resistance mechanism in cocoa and will be a foundation for future researches on this topic. All

the genotypes identified with resistance were used as parental material in establishing polyclonal garden. Planting material from this garden supplied to farmers will ensure tea mosquito bug resistance to certain extent.

Acknowledgments

The authors thank Mondelez India Pvt. Ltd (Formerly known as Cadbury India Ltd.) and Kerala Agricultural University for their assistance to complete the research work.

References

1. Entwistle PF. Pests of cocoa. 1972.
2. Malhotra SK, Apshara SE. Genetic resources of cocoa (Theobroma cacao L.) and their utilizationAn Appraisal. The Indian Journal of Genetics and Plant Breeding.;77(2).
3. Miller NC. Insects associated with cocoa (Theobroma cacao) in Malaya. Bulletin of entomological Research. 1941 Apr; 32(1):1-5.
4. Tan GS. Helopeltis theivora theobromae on cocoa in Malaysia. II. Damage and control. Malaysian Agricultural Research. 1974;3(3):204-12.
5. N'Guessan KF, N'Goran JA, Eskes AB. Mirid resistance studies in Côte d'Ivoire: assessment of antixenosis, antibiosis and tolerance. Global Approaches to Cocoa Germplasm Utilization and Conservation. 2006;(50):177.
6. Lattanzio V, Lattanzio VM, Cardinali A. Role of phenolics in the resistance mechanisms of plants against fungal pathogens and insects. Phytochemistry: Advances in research. 2006;661(2):23-67.
7. Sundararaju D, John JN. Mass rearing technique for *Helopeltis antonii* Sign.(Heteroptera: Miridae)-An important pest of cashew. Journal of Plantation Crops. 1992;20:46.

8. Srikumar KK, Bhat PS. Demographic parameters of *Helopeltis antonii* Signoret (Heteroptera: Miridae) on neem, cocoa and henna. African Journal of Agricultural Research. 2013 Sep 12;8(35):4466-73.
9. Malick CP, Singh MB. Phenolics. Plant enzymology and histoenzymology. Kalyani Publishers, New Delhi. 1980;286.
10. Saroj PL, Bhat PS, Srikumar KK. Tea mosquito bug (*Helopeltis* spp.)–A devastating pest of cashew plantations in India: A review. Indian Journal of Agricultural Sciences. 2016 Feb 1;86(2):151-62.
11. Awang A, Muhamad R, Chong KK. Comparative merits of cocoa pod and shoot as food sources of the mirid, Helopeltis theobromae Miller. Planter. 1988;64(744):100-4.
12. Roy S, Muraleedharan N, Mukhapadhyay A, Handique G. The tea mosquito bug, *Helopeltis theivora* Waterhouse (Heteroptera: Miridae): its status, biology, ecology and management in tea plantations. International Journal of Pest Management. 2015 Jul 3;61(3):179-97.
13. Barakat A, Bagniewska-Zadworna A, Frost CJ, Carlson JE. Phylogeny and expression profiling of CAD and CAD-like genes in hybrid Populus (*P. deltoides*× *P. nigra*): evidence from herbivore damage for subfunctionalization and functional divergence. BMC plant biology. 2010 Dec;10(1):1-1.
14. Van Sumere CF, Albrecht J, Dedonder A, De Pooter H, Pe I. Plant proteins and phenolics. Ch. 8. The Chemistry and Biochemistry of Plant Proteins. JB Harborne, and CF Van Sumere, eds. 1975; 11:211-64.
15. Chakraborty U, Chakraborty N. Impact of environmental factors on infestation of tea leaves by Helopeltis theivora, and associated changes in flavonoid flavor components and enzyme activities. Phytoparasitica. 2005 Mar;33(1):88-96.

16. Bindu N. Die-back in cashew incited by the tea mosquito bug, *Helopeltis antonii* Signoret as influenced by wound pathogens and plant biochemicals (Doctoral dissertation, Department of Agricultural Entomology, College of Horticulture, Vellanikkara).
17. Sundararaju D, Babu PC. Oviposition and feeding deterrency in the matured shoots of cashew and neem against neem mosquito bug, Helopeltis antonii Signoret (Heteroptera: Miridae). Journal of Entomological Research. 2000;24(2):103-7.
18. Nimisha T, Deepthy KB, Subramanian M, Chellappan M, Smitha MS, Girija T. Modulation in certain biochemical constituents of cashew as influenced by tea mosquito bug Helopeltis antonii. Indian Journal of Entomology. 2019;81(3):472-6.
19. Kong LD, Cheng CH, Tan RX. Inhibition of MAO A and B by some plant-derived alkaloids, phenols and anthraquinones. Journal of ethnopharmacology. 2004 Apr 1;91(2-3):351-5.
20. Omokolo ND, Boudjeko T, Whitehead CS. Comparative analyses of alterations in carbohydrates, amino acids, phenols and lignin in roots of three cultivars of *Xanthosoma sagittifolium* infected by *Pythium myriotylum*. South African Journal of Botany. 2005 Nov 1;71(3-4):432-40.

Figure

Click here to access/download;Figure;1.tif

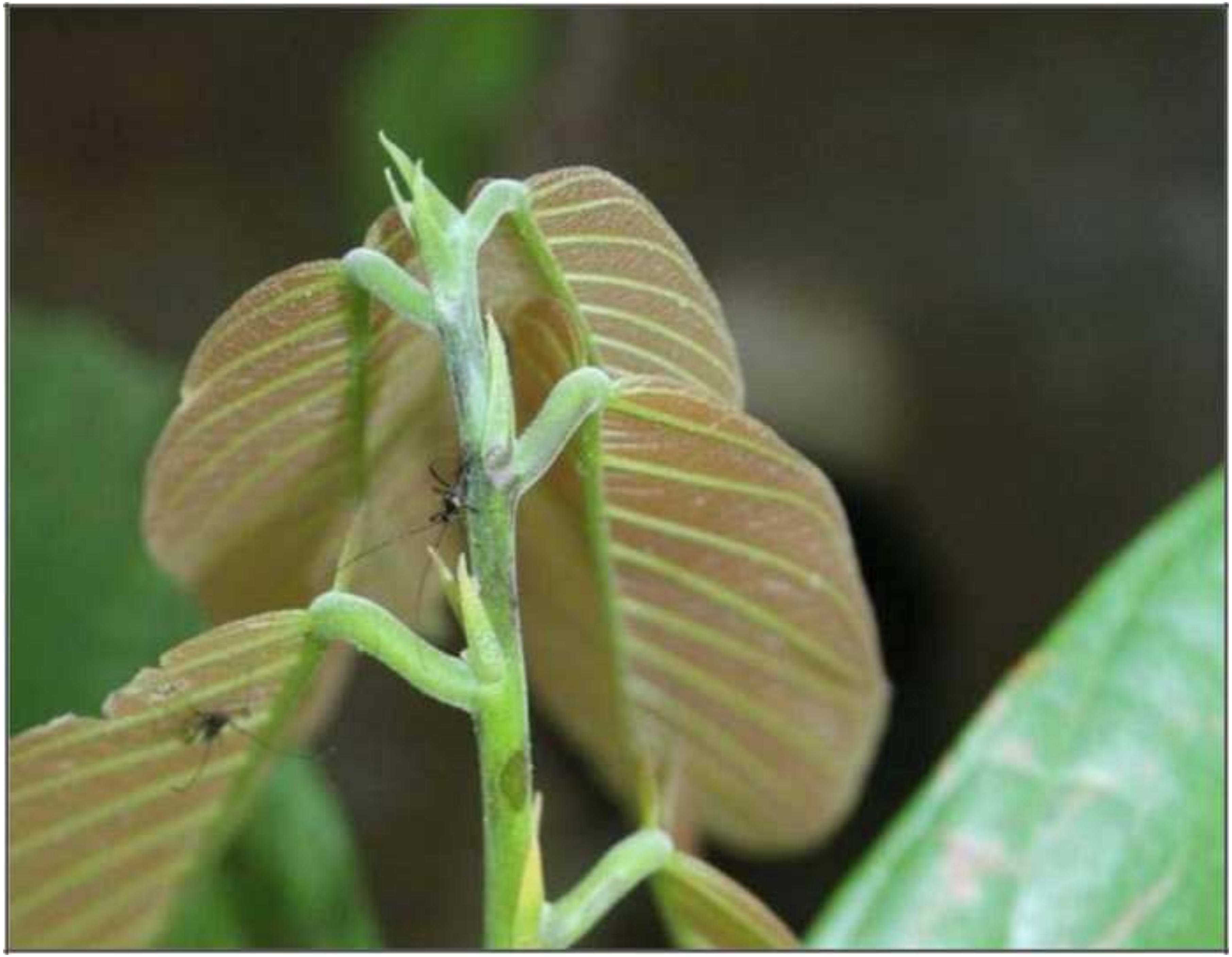

Click here to access/download;Figure;2 (4).tif

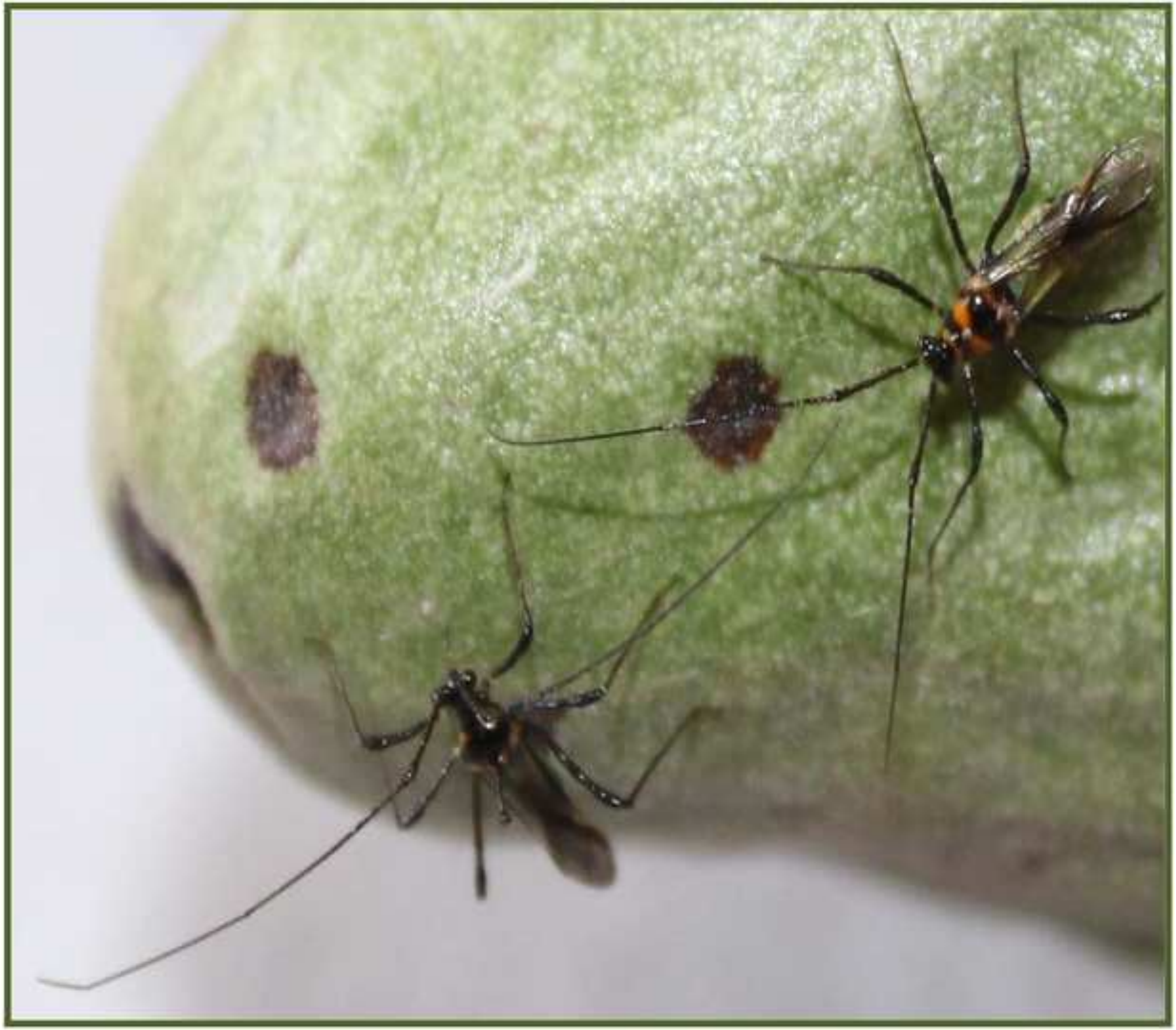

Supporting Information

Click here to access/download
Supporting Information
Additional informations.docx
