# Supplementary material for: Deciphering the role of polyphenol in defence mechanism against tea mosquito bug (Helopeltis theivora Waterhouse.) in cocoa (Theobroma cocoa L.)
Source: PLoS One. 2022 Oct 14;17(10):e0271432. doi: 10.1371/journal.pone.0271432 (PMC9565741; doi:10.1371/journal.pone.0271432)
Supplement: Attachment — Submitted filename: Response to Reviewers.docx [file pone.0271432.s004.docx]

Rebuttal letter

Response to reviewers

PONE-D-22-07611

Deciphering the role of polyphenol in defence mechanism against Tea mosquito bug (*Helopeltis theivora* Waterhouse.) in cocoa (*Theobroma cocoa* L.)

We are thankful to the editors and reviewers for considering our manuscript. In this rebuttal letter we provide responses to all comments raised. Our responses are highlighted with yellow colour and the comments or questions are marked green. We copied the review part from the e-mail and used the same to write our responses.

PONE-D-22-07611

Deciphering the role of polyphenol in defence mechanism against Tea mosquito bug (*Helopeltis theivora* Waterhouse.) in cocoa (*Theobroma cocoa* L.)

PLOS ONE

Dear Dr. JS,

Thank you for submitting your manuscript to PLOS ONE. After careful consideration, we feel that it has merit but does not fully meet PLOS ONE’s publication criteria as it currently stands. Therefore, we invite you to submit a revised version of the manuscript that addresses the points raised during the review process.

Your manuscript has now been reviewed, and the reviewers' comments are appended below. You will see that, while they find your work of interest, they have raised points that need to be addressed before we can make a decision on publication. Pay attention to statistic data, trying to show them clearly.

Please submit your revised manuscript by Jun 09 2022 11:59PM. If you will need more time than this to complete your revisions, please reply to this message or contact the journal office at [plosone@plos.org](mailto:plosone@plos.org). When you're ready to submit your revision, log on to <https://www.editorialmanager.com/pone/> and select the 'Submissions Needing Revision' folder to locate your manuscript file.

Please include the following items when submitting your revised manuscript:

- A rebuttal letter that responds to each point raised by the academic editor and reviewer(s). You should upload this letter as a separate file labeled 'Response to Reviewers'.
- A marked-up copy of your manuscript that highlights changes made to the original version. You should upload this as a separate file labeled 'Revised Manuscript with Track Changes'.
- An unmarked version of your revised paper without tracked changes. You should upload this as a separate file labeled 'Manuscript'.

If you would like to make changes to your financial disclosure, please include your updated statement in your cover letter. Guidelines for resubmitting your figure files are available below the reviewer comments at the end of this letter.

If applicable, we recommend that you deposit your laboratory protocols in [protocols.io](http://protocols.io/) to enhance the reproducibility of your results. Protocols.io assigns your protocol its own identifier (DOI) so that it can be cited independently in the future. For instructions see: <https://journals.plos.org/plosone/s/submission-guidelines#loc-laboratory-protocols>. Additionally, PLOS ONE offers an option for publishing peer-reviewed Lab Protocol articles, which describe protocols hosted on [protocols.io](http://protocols.io/). Read more information on sharing protocols at <https://plos.org/protocols?utm_medium=editorial-email&utm_source=authorletters&utm_campaign=protocols>.

We look forward to receiving your revised manuscript.

Kind regards,

Patrizia Falabella
Academic Editor
PLOS ONE

Journal Requirements:

When submitting your revision, we need you to address these additional requirements.

1. Please ensure that your manuscript meets PLOS ONE's style requirements, including those for file naming. The PLOS ONE style templates can be found at 
<https://journals.plos.org/plosone/s/file?id=wjVg/PLOSOne_formatting_sample_main_body.pdf> and <https://journals.plos.org/plosone/s/file?id=ba62/PLOSOne_formatting_sample_title_authors_affiliations.pdf>

2. We suggest you thoroughly copyedit your manuscript for language usage, spelling, and grammar. If you do not know anyone who can help you do this, you may wish to consider employing a professional scientific editing service.

Whilst you may use any professional scientific editing service of your choice, PLOS has partnered with both American Journal Experts (AJE) and Editage to provide discounted services to PLOS authors. Both organizations have experience helping authors meet PLOS guidelines and can provide language editing, translation, manuscript formatting, and figure formatting to ensure your manuscript meets our submission guidelines. To take advantage of our partnership with AJE, visit the AJE website (<http://learn.aje.com/plos/>) for a 15% discount off AJE services. To take advantage of our partnership with Editage, visit the Editage website ([www.editage.com](http://www.editage.com/)) and enter referral code PLOSEDIT for a 15% discount off Editage services.  If the PLOS editorial team finds any language issues in text that either AJE or Editage has edited, the service provider will re-edit the text for free.

Upon resubmission, please provide the following:
 
The name of the colleague or the details of the professional service that edited your manuscript

A copy of your manuscript showing your changes by either highlighting them or using track changes (uploaded as a *supporting information* file)

A clean copy of the edited manuscript (uploaded as the new *manuscript* file)

3. Please update your submission to use the PLOS LaTeX template. The template and more information on our requirements for LaTeX submissions can be found at <http://journals.plos.org/plosone/s/latex>.

4. We note that the grant information you provided in the ‘Funding Information’ and ‘Financial Disclosure’ sections do not match.

When you resubmit, please ensure that you provide the correct grant numbers for the awards you received for your study in the ‘Funding Information’ section.

5. We note that you have stated that you will provide repository information for your data at acceptance. Should your manuscript be accepted for publication, we will hold it until you provide the relevant accession numbers or DOIs necessary to access your data. If you wish to make changes to your Data Availability statement, please describe these changes in your cover letter and we will update your Data Availability statement to reflect the information you provide.

6. Please include captions for your Supporting Information files at the end of your manuscript, and update any in-text citations to match accordingly. Please see our Supporting Information guidelines for more information: [http://journals.plos.org/plosone/s/supporting-information](http://journals.plos.org/plosone/s/supporting-information).

[Note: HTML markup is below. Please do not edit.]

Reviewers' comments:

Reviewer's Responses to Questions

**Comments to the Author**

1. Is the manuscript technically sound, and do the data support the conclusions?

The manuscript must describe a technically sound piece of scientific research with data that supports the conclusions. Experiments must have been conducted rigorously, with appropriate controls, replication, and sample sizes. The conclusions must be drawn appropriately based on the data presented.

Reviewer #1: Yes

Reviewer #2: Yes

2. Has the statistical analysis been performed appropriately and rigorously?

Reviewer #1: Yes

Reviewer #2: Yes

3. Have the authors made all data underlying the findings in their manuscript fully available?

The [PLOS Data policy](http://www.plosone.org/static/policies.action#sharing) requires authors to make all data underlying the findings described in their manuscript fully available without restriction, with rare exception (please refer to the Data Availability Statement in the manuscript PDF file). The data should be provided as part of the manuscript or its supporting information, or deposited to a public repository. For example, in addition to summary statistics, the data points behind means, medians and variance measures should be available. If there are restrictions on publicly sharing data—e.g. participant privacy or use of data from a third party—those must be specified.

Reviewer #1: Yes

Reviewer #2: Yes

4. Is the manuscript presented in an intelligible fashion and written in standard English?

PLOS ONE does not copyedit accepted manuscripts, so the language in submitted articles must be clear, correct, and unambiguous. Any typographical or grammatical errors should be corrected at revision, so please note any specific errors here.

Reviewer #1: Yes

Reviewer #2: Yes

5. Review Comments to the Author

Please use the space provided to explain your answers to the questions above. You may also include additional comments for the author, including concerns about dual publication, research ethics, or publication ethics. (Please upload your review as an attachment if it exceeds 20,000 characters)

Reviewer #1: Manuscript is well written with new information but the manuscript miss the detailed study.
Comment 1. Rearing of Helopeltis in cocoa pods has to be tried

Response: We tried rearing Helopeltis in cocoa pods and the result was a failure. We are rearing Helopeltis in insect rearing cages for that detached pods were used, these pods start to decay within 4 to 5 days which makes it difficult to rear Helopeltis on cocoa pods.
Comment 2. Screening of cocoa pods and shoots has to be conducted with three species of Helopeltis

Respone: In the cocoa farm of Kerala Agricultural University we have identified only *Helopeltis theivora* attacking cocoa. Even though the adjacent farm is of cashew there we can see all the three species of tea mosquito bug, other two species are not preferring cocoa over cashew. Once we collected all the three species and tried to feed cocoa by enclosing them in insect rearing cages. However, only *Helopeltis theivora* survived.
Comment 3. Cocoa pod screening has to be conducted directly in field to understand the feeding preference

Respone: We conducted field screening during the peak season of attack for consecutive three years and found that the result is on par with the present study.
Comment 4. Choice test has to be conducted

Respone: Budded plants were screened by following choice test. However, it is difficult to collect pods of similar maturity from all the twenty hybrids at a time force test was followed for screening pods.
Comment 5. Helopeltis can easily adapt to resistant clones in matter of time

Respone: Clones with diverse genetic origin is included in the study and all the resistant clones will be used as parents in the establishment of polyclonal garden
Comment 6. All the comments are directly marked in the MS

Respone: Thank you for the effort, we considered all those comments to improve our article

In case of comment, number of generations of rearing: we collected the adult male and female from field, allowed to lay eggs and from that the adults emerged were collected and used for screening. Hence only one generation is reared.
Comment 7. Study has to conducted with above points to get the real answer of resistant clones

Respone: Apart from polyphenol content in case of seedlings colour of the tender flush leaves was also studied and we tried to correlate that with TMB incidence. Unfortunately, we were not able to get a strong correlation. Similarly for pods we also tried to correlate pod shape, unripe pod colour, pod apex and base form and pod rugosity with TMB attack, however none of these characters showed a significant correlation

Reviewer #2: Dear Authors
The work done on screening 20 varieties of cocoa against TMB based on the phenolic activity is appreciated. The findings in this study will definitely help researchers in plant improvement work in future. The manuscript structure is good but author has to be little bit focussed and concentrated while preparing manuscript. Some points for improvement are listed here:
Comment 1. Line 80- the normal reference style is deviated

Respone: The reference styles are now made consistent.
Comment 2. A scoring method to classify the varieties as highly resistant to highly susceptible and reference quoted as no. 8. I had gone through the original paper of Srikumar and Bhatt but couldn’t find any mention on scoring there. They describe life table studies (line 80,151)

Respone: We referred the research article of Srikumar and Bhatt and personally discussed with the author to construct a scoring table which suits our study.
Comment 3. In many places in manuscript, author made non sensible reference citation (line 70,90, 135, 137, 189) It is suggested to go through different papers published in PloS One journal for understanding the correct citation of references in text.

Respone: Sorry for the mistake from our part, references are now made consistent with PloS One format
Comment 4. Line 101 and 102-repetition

Respone: We removed the repeated line from the manuscript
Comment 5. It is good if you can add author name to the scientific name when mentioned first time in text (line 130 for Mikania micrantha)

Respone: We made it correct in the manuscript by adding authors name
Comment 6. Line 131- females started laying eggs in two days… whether they were collected from field or from insectary?

Respone: Adult males and females were collected from field and allowed to mate and lay eggs
Comment 7. In Line 87 Fig 2 is mentioned which is quite irrelevant

Respone: We removed it from the manuscript
Comment 8. Results portion may be modified and thoroughly checked as some mismatching were found. Line 151- author says 5 hybrids were ranked as Highly resistant where as table shows there were seven. Listing of varieties based on your observations in text should not be confusing to the readers. Please make it in order as they appear or in ascending /descending order of resistant.

Respone: Sorry for the inconvenience, we modified the result portion and the table in the manuscript.
Comment 9. A strong negative correlation is not observed especially in case of pods

Respone: A moderate negative correlation was observed in case of pod. Necessary corrections are made in the manuscript.
Comment 10. Please pay more attention and ethics while preparing manuscript in future

Respone: We sincerely appreciate all valuable comments which helped used to improve our article. We have revised the article as per the suggestion of reviewers. Surely, we will pay more attention while writing the article in the future.

6. PLOS authors have the option to publish the peer review history of their article ([what does this mean?](https://journals.plos.org/plosone/s/editorial-and-peer-review-process#loc-peer-review-history)). If published, this will include your full peer review and any attached files.


If you choose “no”, your identity will remain anonymous but your review may still be made public.


**Do you want your identity to be public for this peer review?** For information about this choice, including consent withdrawal, please see our [Privacy Policy](https://www.plos.org/privacy-policy).
