# Supplementary material for: Deciphering the role of polyphenol in defence mechanism against tea mosquito bug (Helopeltis theivora Waterhouse.) in cocoa (Theobroma cocoa L.)
Source: PLoS One. 2022 Oct 14;17(10):e0271432. doi: 10.1371/journal.pone.0271432 (PMC9565741; doi:10.1371/journal.pone.0271432)
Supplement: Attachment — Submitted filename: plos one 2022 second revision.docx [file pone.0271432.s005.docx]

Dear Editor

Happy to know that almost all the suggestions which I pointed out were taken seriously by the authors and found incorporated in the revised manuscript. But still some points have to be addressed before accepting the same for publishing. The points are mentioned below:

1. Scoring method on screening is still not conspicuous. Not satisfied with the explanation by the authors. Reference mentioned in line 87 doesn’t pertain to the subject (scoring). Authors replied that they discussed with the authors of reference cited (as ref. no. 8) and derived a method. Developing a reliable scoring method is fine but citing an irrelevant reference is not a good practice.
2. What is the transformation used for data in table 3? It is not mentioned in table or text.
3. Reference 16 is not in journal format. please refer <https://journals.plos.org/plosone/s/submission-guidelines> for assistance
4. Year of publication and page no. are missing from reference no. 2. The correct year is 2017 and page no.199-213.
