# Supplementary material for: Deciphering the role of polyphenol in defence mechanism against tea mosquito bug (Helopeltis theivora Waterhouse.) in cocoa (Theobroma cocoa L.)
Source: PLoS One. 2022 Oct 14;17(10):e0271432. doi: 10.1371/journal.pone.0271432 (PMC9565741; doi:10.1371/journal.pone.0271432)
Supplement: Attachment — Submitted filename: Response to Reviewers.docx [file pone.0271432.s006.docx]

Rebuttal letter

Response to reviewers

PONE-D-22-07611

Deciphering the role of polyphenol in defence mechanism against Tea mosquito bug (*Helopeltis theivora* Waterhouse.) in cocoa (*Theobroma cocoa* L.)

We are thankful to the editor and reviewers for considering our manuscript. We modified the manuscript to address the points made by the editor and the reviewers. In this rebuttal letter, we provide responses to all comments raised. We are sorry to inform you that we have made a mistake in writing the affiliation of an author in the title page and it has been corrected. Please, consider this correction also. Our responses are highlighted with yellow colour and the comments or questions are marked green. We copied the review comments to author part from the e-mail and used the same to write our responses.

Review Comments to the Author

Please use the space provided to explain your answers to the questions above. You may also include additional comments for the author, including concerns about dual publication, research ethics, or publication ethics. (Please upload your review as an attachment if it exceeds 20,000 characters)

Reviewer #2: Happy to know that almost all the suggestions which I pointed out were taken seriously by the authors and found incorporated in the revised manuscript. But still some points have to be addressed before accepting the same for publishing. The points are mentioned below:

Response: Thank you. We appreciate the time and effort that you have dedicated to providing your valuable feedback on the manuscript. We sincerely appreciate all valuable comments which helped us to improve our article. We tried to incorporate changes to reflect most of the suggestions provided. We have revised the article as per the suggestions.
Comment 1. Scoring method on screening is still not conspicuous. Not satisfied with the explanation by the authors. Reference mentioned in line 87 doesn’t pertain to the subject (scoring). Authors replied that they discussed with the authors of reference cited (as ref. no. 8) and derived a method. Developing a reliable scoring method is fine but citing an irrelevant reference is not a good practice.

Response: Thank you for pointing this out and sorry for such a mistake from our part. Surely, we will pay more attention while writing the article in the future. We decided to remove that reference from the manuscript. We have incorporated our thesis reference stating that the chart is prepared by us. We really apologize for our mistake and promise not to repeat the same.
Comment 2. What is the transformation used for data in table 3? It is not mentioned in table or text.

Response: Angular transformation was used and necessary corrections are made in the manuscript.
Comment 3. Reference 16 is not in journal format. please refer <https://journals.plos.org/plosone/s/submission-guidelines> for assistance

Response: Sorry for the mistake from our part, references are now made consistent with PloS One format
Comment 4. Year of publication and page no. are missing from reference no. 2. The correct year is 2017 and page no.199-213.

Response: Sorry for the mistake, year and page number have been added in reference no. 2. in the manuscript.
