# Supplementary material for: Deciphering the role of polyphenol in defence mechanism against tea mosquito bug (Helopeltis theivora Waterhouse.) in cocoa (Theobroma cocoa L.)
Source: PLoS One. 2022 Oct 14;17(10):e0271432. doi: 10.1371/journal.pone.0271432 (PMC9565741; doi:10.1371/journal.pone.0271432)
Supplement: Peer review history

# Peer review: Decision Letter 0

**DOI:** 10.1371/journal.pone.0271432.r001

**Authors:** Patrizia Falabella

**Transfer Alert**

This paper was transferred from another journal. As a result, its full editorial history (including decision letters, peer reviews and author responses) may not be present.

26 Apr 2022

PONE-D-22-07611Deciphering the role of polyphenol in defence mechanism against Tea mosquito bug (Helopeltis theivora Waterhouse.) in cocoa (Theobroma cocoa L.)PLOS ONE

Dear Dr. JS,

Thank you for submitting your manuscript to PLOS ONE. After careful consideration, we feel that it has merit but does not fully meet PLOS ONE’s publication criteria as it currently stands. Therefore, we invite you to submit a revised version of the manuscript that addresses the points raised during the review process.

Your manuscript has now been reviewed, and the reviewers' comments are appended below. You will see that, while they find your work of interest, they have raised points that need to be addressed before we can make a decision on publication. Pay attention to statistic data, trying to show them clearly.

Please submit your revised manuscript by Jun 09 2022 11:59PM. If you will need more time than this to complete your revisions, please reply to this message or contact the journal office at plosone@plos.org. When you're ready to submit your revision, log on to https://www.editorialmanager.com/pone/ and select the 'Submissions Needing Revision' folder to locate your manuscript file.

Please include the following items when submitting your revised manuscript:

- A rebuttal letter that responds to each point raised by the academic editor and reviewer(s). You should upload this letter as a separate file labeled 'Response to Reviewers'.
- A marked-up copy of your manuscript that highlights changes made to the original version. You should upload this as a separate file labeled 'Revised Manuscript with Track Changes'.
- An unmarked version of your revised paper without tracked changes. You should upload this as a separate file labeled 'Manuscript'.

If you would like to make changes to your financial disclosure, please include your updated statement in your cover letter. Guidelines for resubmitting your figure files are available below the reviewer comments at the end of this letter.

If applicable, we recommend that you deposit your laboratory protocols in protocols.io to enhance the reproducibility of your results. Protocols.io assigns your protocol its own identifier (DOI) so that it can be cited independently in the future. For instructions see: https://journals.plos.org/plosone/s/submission-guidelines#loc-laboratory-protocols. Additionally, PLOS ONE offers an option for publishing peer-reviewed Lab Protocol articles, which describe protocols hosted on protocols.io. Read more information on sharing protocols at https://plos.org/protocols?utm_medium=editorial-email&utm_source=authorletters&utm_campaign=protocols.

We look forward to receiving your revised manuscript.

Kind regards,

Patrizia Falabella

Academic Editor

PLOS ONE

Journal Requirements:

When submitting your revision, we need you to address these additional requirements.

1. Please ensure that your manuscript meets PLOS ONE's style requirements, including those for file naming. The PLOS ONE style templates can be found at 

https://journals.plos.org/plosone/s/file?id=wjVg/PLOSOne_formatting_sample_main_body.pdf and https://journals.plos.org/plosone/s/file?id=ba62/PLOSOne_formatting_sample_title_authors_affiliations.pdf

2. We suggest you thoroughly copyedit your manuscript for language usage, spelling, and grammar. If you do not know anyone who can help you do this, you may wish to consider employing a professional scientific editing service. 

Whilst you may use any professional scientific editing service of your choice, PLOS has partnered with both American Journal Experts (AJE) and Editage to provide discounted services to PLOS authors. Both organizations have experience helping authors meet PLOS guidelines and can provide language editing, translation, manuscript formatting, and figure formatting to ensure your manuscript meets our submission guidelines. To take advantage of our partnership with AJE, visit the AJE website (http://learn.aje.com/plos/) for a 15% discount off AJE services. To take advantage of our partnership with Editage, visit the Editage website (www.editage.com) and enter referral code PLOSEDIT for a 15% discount off Editage services.  If the PLOS editorial team finds any language issues in text that either AJE or Editage has edited, the service provider will re-edit the text for free.

Upon resubmission, please provide the following:

The name of the colleague or the details of the professional service that edited your manuscript

A copy of your manuscript showing your changes by either highlighting them or using track changes (uploaded as a *supporting information* file)

A clean copy of the edited manuscript (uploaded as the new *manuscript* file)

3. Please update your submission to use the PLOS LaTeX template. The template and more information on our requirements for LaTeX submissions can be found at http://journals.plos.org/plosone/s/latex.

4. We note that the grant information you provided in the ‘Funding Information’ and ‘Financial Disclosure’ sections do not match. 

When you resubmit, please ensure that you provide the correct grant numbers for the awards you received for your study in the ‘Funding Information’ section.

5. We note that you have stated that you will provide repository information for your data at acceptance. Should your manuscript be accepted for publication, we will hold it until you provide the relevant accession numbers or DOIs necessary to access your data. If you wish to make changes to your Data Availability statement, please describe these changes in your cover letter and we will update your Data Availability statement to reflect the information you provide.

6. Please include captions for your Supporting Information files at the end of your manuscript, and update any in-text citations to match accordingly. Please see our Supporting Information guidelines for more information: http://journals.plos.org/plosone/s/supporting-information.

[Note: HTML markup is below. Please do not edit.]

Reviewers' comments:

Reviewer's Responses to Questions

**Comments to the Author**

1. Is the manuscript technically sound, and do the data support the conclusions?

The manuscript must describe a technically sound piece of scientific research with data that supports the conclusions. Experiments must have been conducted rigorously, with appropriate controls, replication, and sample sizes. The conclusions must be drawn appropriately based on the data presented. 

Reviewer #1: Yes

Reviewer #2: Yes

**********

2. Has the statistical analysis been performed appropriately and rigorously? 

Reviewer #1: Yes

Reviewer #2: Yes

**********

3. Have the authors made all data underlying the findings in their manuscript fully available?

The PLOS Data policy requires authors to make all data underlying the findings described in their manuscript fully available without restriction, with rare exception (please refer to the Data Availability Statement in the manuscript PDF file). The data should be provided as part of the manuscript or its supporting information, or deposited to a public repository. For example, in addition to summary statistics, the data points behind means, medians and variance measures should be available. If there are restrictions on publicly sharing data—e.g. participant privacy or use of data from a third party—those must be specified.

Reviewer #1: Yes

Reviewer #2: Yes

**********

4. Is the manuscript presented in an intelligible fashion and written in standard English?

PLOS ONE does not copyedit accepted manuscripts, so the language in submitted articles must be clear, correct, and unambiguous. Any typographical or grammatical errors should be corrected at revision, so please note any specific errors here.

Reviewer #1: Yes

Reviewer #2: Yes

**********

5. Review Comments to the Author

Please use the space provided to explain your answers to the questions above. You may also include additional comments for the author, including concerns about dual publication, research ethics, or publication ethics. (Please upload your review as an attachment if it exceeds 20,000 characters)

Reviewer #1: Manuscript is well written with new information but the manuscript miss the detailed study.

1. Rearing of Helopeltis in cocoa pods has to be tried

2. Screening of cocoa pods and shoots has to be conducted with three species of Helopeltis

3. Cocoa pod screening has to be condcuted directly in field to understand the feeding preference

4. Choice test has to be conducted

5. Helopeltis can easily adapt to resistant clones in matter of time

6. All the comments are directly marked in the MS

7. Study has to conducted with above points to get the real answer of resistant clones

Reviewer #2: Dear Authors

The work done on screening 20 varieties of cocoa against TMB based on the phenolic activity is appreciated. The findings in this study will definitely help researchers in plant improvement work in future. The manuscript structure is good but author has to be little bit focussed and concentrated while preparing manuscript. Some points for improvement are listed here:

1. Line 80- the normal reference style is deviated

2. A scoring method to classify the varieties as highly resistant to highly susceptible and reference quoted as no. 8. I had gone through the original paper of Srikumar and Bhatt but couldn’t find any mention on scoring there. They describe life table studies (line 80,151)

3. In many places in manuscript, author made non sensible reference citation ( line 70,90, 135, 137, 189) It is suggested to go through different papers published in PloS One journal for understanding the correct citation of references in text.

4. Line 101 and 102-repetition

5. It is good if you can add author name to the scientific name when mentioned first time in text ( line 130 for Mikania micrantha)

6. Line 131- females started laying eggs in two days… whether they were collected from field or from insectary?

7. In Line 87 Fig 2 is mentioned which is quite irrelevant

8. Results portion may be modified and thoroughly checked as some mismatching were found. Line 151- author says 5 hybrids were ranked as Highly resistant where as table shows there were seven. Listing of varieties based on your observations in text should not be confusing to the readers. Please make it in order as they appear or in ascending /descending order of resistant.

9. A strong negative correlation is not observed especially in case of pods

10. Please pay more attention and ethics while preparing manuscript in future

**********

6. PLOS authors have the option to publish the peer review history of their article (what does this mean?). If published, this will include your full peer review and any attached files.

If you choose “no”, your identity will remain anonymous but your review may still be made public.

**Do you want your identity to be public for this peer review?** For information about this choice, including consent withdrawal, please see our Privacy Policy.

Reviewer #1: **Yes: **SRIKUMAR KODAKKADAN

Reviewer #2: No

[NOTE: If reviewer comments were submitted as an attachment file, they will be attached to this email and accessible via the submission site. Please log into your account, locate the manuscript record, and check for the action link "View Attachments". If this link does not appear, there are no attachment files.]

While revising your submission, please upload your figure files to the Preflight Analysis and Conversion Engine (PACE) digital diagnostic tool, https://pacev2.apexcovantage.com/. PACE helps ensure that figures meet PLOS requirements. To use PACE, you must first register as a user. Registration is free. Then, login and navigate to the UPLOAD tab, where you will find detailed instructions on how to use the tool. If you encounter any issues or have any questions when using PACE, please email PLOS at figures@plos.org. Please note that Supporting Information files do not need this step.

Attachment Submitted filename: PONE-D-22-07611.pdf Click here for additional data file.

# Author comment: Author response to Decision Letter 0

**DOI:** 10.1371/journal.pone.0271432.r002

30 May 2022

Comment 1. Rearing of Helopeltis in cocoa pods has to be tried

Response: We tried rearing Helopeltis in cocoa pods and the result was a failure. We are rearing Helopeltis in insect rearing cages for that detached pods were used, these pods start to decay within 4 to 5 days which makes it difficult to rear Helopeltis on cocoa pods.

Comment 2. Screening of cocoa pods and shoots has to be conducted with three species of Helopeltis

Respone: In the cocoa farm of Kerala Agricultural University we have identified only Helopeltis theivora attacking cocoa. Even though the adjacent farm is of cashew there we can see all the three species of tea mosquito bug, other two species are not preferring cocoa over cashew. Once we collected all the three species and tried to feed cocoa by enclosing them in insect rearing cages. However, only Helopeltis theivora survived.

Comment 3. Cocoa pod screening has to be conducted directly in field to understand the feeding preference

Respone: We conducted field screening during the peak season of attack for consecutive three years and found that the result is on par with the present study.

Comment 4. Choice test has to be conducted

Respone: Budded plants were screened by following choice test. However, it is difficult to collect pods of similar maturity from all the twenty hybrids at a time force test was followed for screening pods.

Comment 5. Helopeltis can easily adapt to resistant clones in matter of time

Respone: Clones with diverse genetic origin is included in the study and all the resistant clones will be used as parents in the establishment of polyclonal garden 

Comment 6. All the comments are directly marked in the MS

Respone: Thank you for the effort, we considered all those comments to improve our article

Comment 7. Study has to conducted with above points to get the real answer of resistant clones

Respone: Apart from polyphenol content in case of seedlings colour of the tender flush leaves was also studied and we tried to correlate that with TMB incidence. Unfortunately, we were not able to get a strong correlation. Similarly for pods we also tried to correlate pod shape, unripe pod colour, pod apex and base form and pod rugosity with TMB attack, however none of these characters showed a significant correlation

Reviewer #2: Dear Authors

The work done on screening 20 varieties of cocoa against TMB based on the phenolic activity is appreciated. The findings in this study will definitely help researchers in plant improvement work in future. The manuscript structure is good but author has to be little bit focussed and concentrated while preparing manuscript. Some points for improvement are listed here:

Comment 1. Line 80- the normal reference style is deviated

Respone: The reference styles are now made consistent.

Comment 2. A scoring method to classify the varieties as highly resistant to highly susceptible and reference quoted as no. 8. I had gone through the original paper of Srikumar and Bhatt but couldn’t find any mention on scoring there. They describe life table studies (line 80,151)

Respone: We referred the research article of Srikumar and Bhatt and discussed with the authors to construct a scoring table which suits our study.

Comment 3. In many places in manuscript, author made non sensible reference citation ( line 70,90, 135, 137, 189) It is suggested to go through different papers published in PloS One journal for understanding the correct citation of references in text.

Respone: Sorry for the mistake from our part, references are now made consistent with PloS One format

Comment 4. Line 101 and 102-repetition

Respone: We removed the repeated line from the manuscript

Comment 5. It is good if you can add author name to the scientific name when mentioned first time in text ( line 130 for Mikania micrantha)

Respone: We made it correct in the manuscript by adding authors name 

Comment 6. Line 131- females started laying eggs in two days… whether they were collected from field or from insectary?

Respone: Adult males and females were collected from field and allowed to mate and lay eggs

Comment 7. In Line 87 Fig 2 is mentioned which is quite irrelevant

Respone: We removed it from the manuscript

Comment 8. Results portion may be modified and thoroughly checked as some mismatching were found. Line 151- author says 5 hybrids were ranked as Highly resistant where as table shows there were seven. Listing of varieties based on your observations in text should not be confusing to the readers. Please make it in order as they appear or in ascending /descending order of resistant.

Respone: Sorry for the inconvenience, we modified the result portion and the table in the manuscript. 

Comment 9. A strong negative correlation is not observed especially in case of pods

Respone: A moderate negative correlation was observed in case of pod. Necessary corrections are made in the manuscript.

Comment 10. Please pay more attention and ethics while preparing manuscript in future

Respone: We sincerely appreciate all valuable comments which helped used to improve our article. We have revised the article as per the suggestion of reviewers. Surely, we will pay more attention while writing the article in the future.

Attachment Submitted filename: Response to Reviewers.docx Click here for additional data file.

# Peer review: Decision Letter 1

**DOI:** 10.1371/journal.pone.0271432.r003

**Authors:** Patrizia Falabella

27 Jun 2022

PONE-D-22-07611R1Deciphering the role of polyphenol in defence mechanism against Tea mosquito bug (Helopeltis theivora Waterhouse.) in cocoa (Theobroma cacao L.)PLOS ONE

Dear Dr. Minimol,

Thank you for submitting your manuscript to PLOS ONE. After careful consideration, we feel that it has merit but does not fully meet PLOS ONE’s publication criteria as it currently stands. Therefore, we invite you to submit a revised version of the manuscript that addresses the points raised during the review process.

Please submit your revised manuscript by 08-07-2022. If you will need more time than this to complete your revisions, please reply to this message or contact the journal office at plosone@plos.org. When you're ready to submit your revision, log on to https://www.editorialmanager.com/pone/ and select the 'Submissions Needing Revision' folder to locate your manuscript file.

Please include the following items when submitting your revised manuscript:

- A rebuttal letter that responds to each point raised by the academic editor and reviewer(s). You should upload this letter as a separate file labeled 'Response to Reviewers'.
- A marked-up copy of your manuscript that highlights changes made to the original version. You should upload this as a separate file labeled 'Revised Manuscript with Track Changes'.
- An unmarked version of your revised paper without tracked changes. You should upload this as a separate file labeled 'Manuscript'.

If you would like to make changes to your financial disclosure, please include your updated statement in your cover letter. Guidelines for resubmitting your figure files are available below the reviewer comments at the end of this letter.

If applicable, we recommend that you deposit your laboratory protocols in protocols.io to enhance the reproducibility of your results. Protocols.io assigns your protocol its own identifier (DOI) so that it can be cited independently in the future. For instructions see: https://journals.plos.org/plosone/s/submission-guidelines#loc-laboratory-protocols. Additionally, PLOS ONE offers an option for publishing peer-reviewed Lab Protocol articles, which describe protocols hosted on protocols.io. Read more information on sharing protocols at https://plos.org/protocols?utm_medium=editorial-email&utm_source=authorletters&utm_campaign=protocols.

We look forward to receiving your revised manuscript.

Kind regards,

Patrizia Falabella

Academic Editor

PLOS ONE

Journal Requirements:

Please review your reference list to ensure that it is complete and correct. If you have cited papers that have been retracted, please include the rationale for doing so in the manuscript text, or remove these references and replace them with relevant current references. Any changes to the reference list should be mentioned in the rebuttal letter that accompanies your revised manuscript. If you need to cite a retracted article, indicate the article’s retracted status in the References list and also include a citation and full reference for the retraction notice.

[Note: HTML markup is below. Please do not edit.]

Reviewers' comments:

Reviewer's Responses to Questions

**Comments to the Author**

1. If the authors have adequately addressed your comments raised in a previous round of review and you feel that this manuscript is now acceptable for publication, you may indicate that here to bypass the “Comments to the Author” section, enter your conflict of interest statement in the “Confidential to Editor” section, and submit your "Accept" recommendation.

Reviewer #2: (No Response)

**********

2. Is the manuscript technically sound, and do the data support the conclusions?

The manuscript must describe a technically sound piece of scientific research with data that supports the conclusions. Experiments must have been conducted rigorously, with appropriate controls, replication, and sample sizes. The conclusions must be drawn appropriately based on the data presented. 

Reviewer #2: Yes

**********

3. Has the statistical analysis been performed appropriately and rigorously? 

Reviewer #2: Yes

**********

4. Have the authors made all data underlying the findings in their manuscript fully available?

The PLOS Data policy requires authors to make all data underlying the findings described in their manuscript fully available without restriction, with rare exception (please refer to the Data Availability Statement in the manuscript PDF file). The data should be provided as part of the manuscript or its supporting information, or deposited to a public repository. For example, in addition to summary statistics, the data points behind means, medians and variance measures should be available. If there are restrictions on publicly sharing data—e.g. participant privacy or use of data from a third party—those must be specified.

Reviewer #2: Yes

**********

5. Is the manuscript presented in an intelligible fashion and written in standard English?

PLOS ONE does not copyedit accepted manuscripts, so the language in submitted articles must be clear, correct, and unambiguous. Any typographical or grammatical errors should be corrected at revision, so please note any specific errors here.

Reviewer #2: Yes

**********

6. Review Comments to the Author

Please use the space provided to explain your answers to the questions above. You may also include additional comments for the author, including concerns about dual publication, research ethics, or publication ethics. (Please upload your review as an attachment if it exceeds 20,000 characters)

Reviewer #2: Happy to know that almost all the suggestions which I pointed out were taken seriously by the authors and found incorporated in the revised manuscript. But still some points have to be addressed before accepting the same for publishing. The points are mentioned below:

1. Scoring method on screening is still not conspicuous. Not satisfied with the explanation by the authors. Reference mentioned in line 87 doesn’t pertain to the subject (scoring). Authors replied that they discussed with the authors of reference cited (as ref. no. 8) and derived a method. Developing a reliable scoring method is fine but citing an irrelevant reference is not a good practice.

2. What is the transformation used for data in table 3? It is not mentioned in table or text.

3. Reference 16 is not in journal format. please refer https://journals.plos.org/plosone/s/submission-guidelines for assistance

4. Year of publication and page no. are missing from reference no. 2. The correct year is 2017 and page no.199-213.

**********

7. PLOS authors have the option to publish the peer review history of their article (what does this mean?). If published, this will include your full peer review and any attached files.

If you choose “no”, your identity will remain anonymous but your review may still be made public.

**Do you want your identity to be public for this peer review?** For information about this choice, including consent withdrawal, please see our Privacy Policy.

Reviewer #2: No

**********

[NOTE: If reviewer comments were submitted as an attachment file, they will be attached to this email and accessible via the submission site. Please log into your account, locate the manuscript record, and check for the action link "View Attachments". If this link does not appear, there are no attachment files.]

While revising your submission, please upload your figure files to the Preflight Analysis and Conversion Engine (PACE) digital diagnostic tool, https://pacev2.apexcovantage.com/. PACE helps ensure that figures meet PLOS requirements. To use PACE, you must first register as a user. Registration is free. Then, login and navigate to the UPLOAD tab, where you will find detailed instructions on how to use the tool. If you encounter any issues or have any questions when using PACE, please email PLOS at figures@plos.org. Please note that Supporting Information files do not need this step.

Attachment Submitted filename: plos one 2022 second revision.docx Click here for additional data file.

# Author comment: Author response to Decision Letter 1

**DOI:** 10.1371/journal.pone.0271432.r004

29 Jun 2022

Review Comments to the Author

Please use the space provided to explain your answers to the questions above. You may also include additional comments for the author, including concerns about dual publication, research ethics, or publication ethics. (Please upload your review as an attachment if it exceeds 20,000 characters)

Reviewer #2: Happy to know that almost all the suggestions which I pointed out were taken seriously by the authors and found incorporated in the revised manuscript. But still some points have to be addressed before accepting the same for publishing. The points are mentioned below:

Response: Thank you. We appreciate the time and effort that you have dedicated to providing your valuable feedback on the manuscript. We sincerely appreciate all valuable comments which helped us to improve our article. We tried to incorporate changes to reflect most of the suggestions provided. We have revised the article as per the suggestions.

Comment 1. Scoring method on screening is still not conspicuous. Not satisfied with the explanation by the authors. Reference mentioned in line 87 doesn’t pertain to the subject (scoring). Authors replied that they discussed with the authors of reference cited (as ref. no. 8) and derived a method. Developing a reliable scoring method is fine but citing an irrelevant reference is not a good practice.

Response: Thank you for pointing this out and sorry for such a mistake from our part. Surely, we will pay more attention while writing the article in the future. We decided to remove that reference from the manuscript. We have incorporated our thesis reference stating that the chart is prepared by us. We really apologize for our mistake and promise not to repeat the same.

Comment 2. What is the transformation used for data in table 3? It is not mentioned in table or text.

Response: Angular transformation was used and necessary corrections are made in the manuscript.

Comment 3. Reference 16 is not in journal format. please refer https://journals.plos.org/plosone/s/submission-guidelines for assistance

Response: Sorry for the mistake from our part, references are now made consistent with PloS One format

Comment 4. Year of publication and page no. are missing from reference no. 2. The correct year is 2017 and page no.199-213.

Response: Sorry for the mistake, year and page number have been added in reference no. 2. in the manuscript.

Attachment Submitted filename: Response to Reviewers.docx Click here for additional data file.

# Editor report: Decision Letter 2

**DOI:** 10.1371/journal.pone.0271432.r005

**Authors:** Patrizia Falabella

1 Jul 2022

Deciphering the role of polyphenol in defence mechanism against Tea mosquito bug (Helopeltis theivora Waterhouse.) in cocoa (Theobroma cacao L.)

PONE-D-22-07611R2

Dear Dr. Minimol,

We’re pleased to inform you that your manuscript has been judged scientifically suitable for publication and will be formally accepted for publication once it meets all outstanding technical requirements.

Within one week, you’ll receive an e-mail detailing the required amendments. When these have been addressed, you’ll receive a formal acceptance letter and your manuscript will be scheduled for publication.

An invoice for payment will follow shortly after the formal acceptance. To ensure an efficient process, please log into Editorial Manager at http://www.editorialmanager.com/pone/, click the 'Update My Information' link at the top of the page, and double check that your user information is up-to-date. If you have any billing related questions, please contact our Author Billing department directly at authorbilling@plos.org.

If your institution or institutions have a press office, please notify them about your upcoming paper to help maximize its impact. If they’ll be preparing press materials, please inform our press team as soon as possible -- no later than 48 hours after receiving the formal acceptance. Your manuscript will remain under strict press embargo until 2 pm Eastern Time on the date of publication. For more information, please contact onepress@plos.org.

Kind regards,

Patrizia Falabella

Academic Editor

PLOS ONE

Additional Editor Comments (optional):

Reviewers' comments:

# Editor report: Acceptance letter

**DOI:** 10.1371/journal.pone.0271432.r006

**Authors:** Patrizia Falabella

23 Sep 2022

PONE-D-22-07611R2 

*Deciphering the role of polyphenol in defence mechanism against Tea mosquito bug (Helopeltis theivora Waterhouse.) in cocoa (Theobroma cocoa L.)*

Dear Dr. J. S.:

I'm pleased to inform you that your manuscript has been deemed suitable for publication in PLOS ONE. Congratulations! Your manuscript is now with our production department. 

If your institution or institutions have a press office, please let them know about your upcoming paper now to help maximize its impact. If they'll be preparing press materials, please inform our press team within the next 48 hours. Your manuscript will remain under strict press embargo until 2 pm Eastern Time on the date of publication. For more information please contact onepress@plos.org.

If we can help with anything else, please email us at plosone@plos.org. 

Thank you for submitting your work to PLOS ONE and supporting open access. 

Kind regards, 

PLOS ONE Editorial Office Staff

on behalf of

Prof. Patrizia Falabella 

Academic Editor

PLOS ONE
